# Supplementary material for: Integration of eQTL and GEO Datasets to Identify Genes Associated with Breast Ductal Carcinoma In Situ
Source: Curr Issues Mol Biol. 2025 Sep 11;47(9):747. doi: 10.3390/cimb47090747 (PMC12468441; doi:10.3390/cimb47090747)
Supplement: Supplementary file 1 [file cimb-47-00747-s001.zip › Supplementary Figures S1.pdf]

Supplemental Figures S1. Visualization charts of mendelian randomization analysis based on 13 differentially expressed genes. (A) forest plots; (B) funnel plots; (C) leave -one-out method analysis plots; and (D) scatter plots.

APOBEC3G:

A

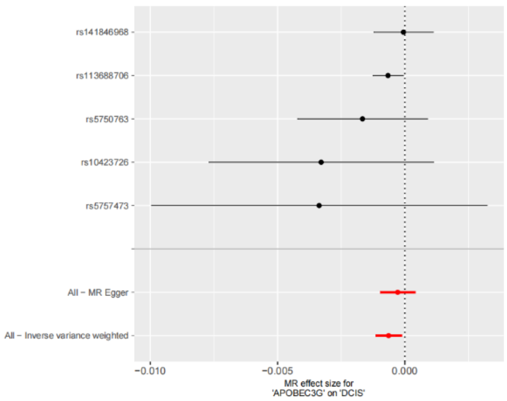

C

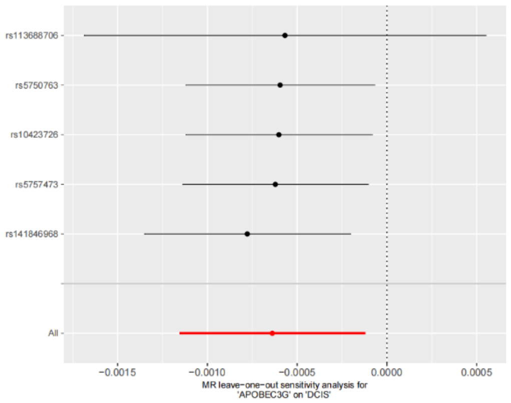

B

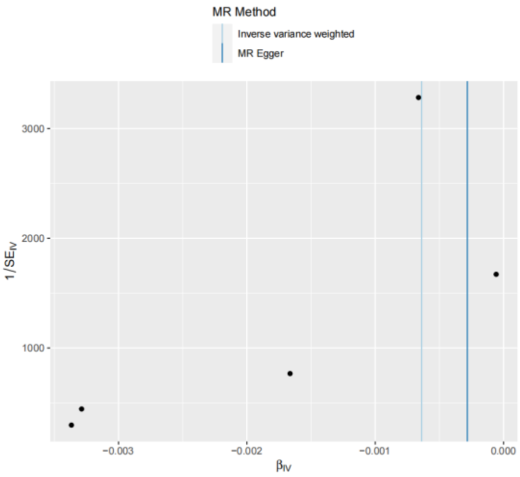

D

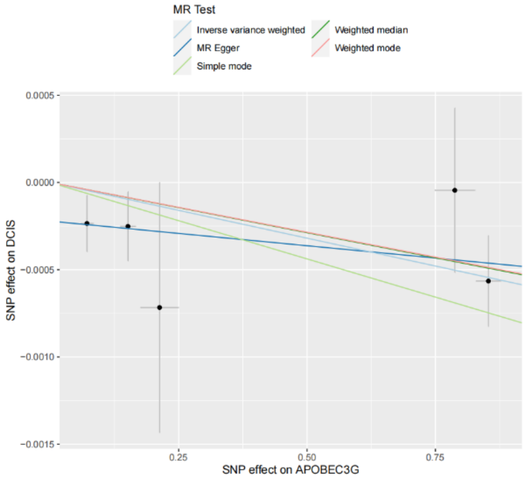

CYB5R2:

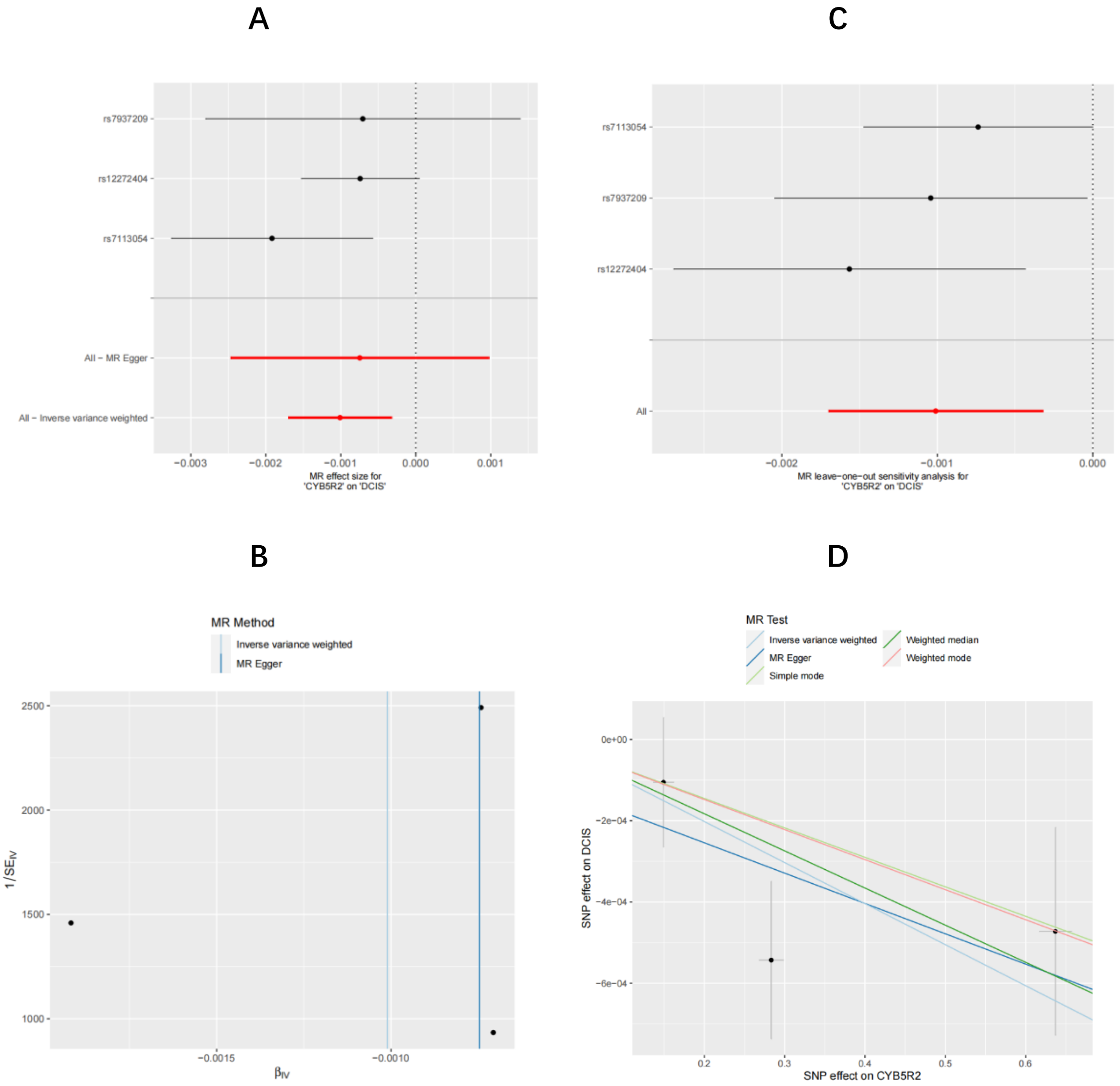

A
rs7937209
rs12272404
rs7113054
All − MR Egger
All − Inverse variance weighted
−0.003
−0.002
−0.001
0.000
0.001
MR effect size for
'CYB5R2' on 'DCIS'
C
rs7113054
rs7937209
rs12272404
All
−0.002
−0.001
0.000
MR leave−one−out sensitivity analysis for
'CYB5R2' on 'DCIS'
B
MR Method
Inverse variance weighted
MR Egger
2500
2000
1500
1000
1/SE_IV
−0.0015
−0.0010
β_IV
D
MR Test
Inverse variance weighted
MR Egger
Simple mode
Weighted median
Weighted mode
0e+00
−2e−04
−4e−04
−6e−04
SNP effect on DCIS
0.2
0.3
0.4
0.5
0.6
SNP effect on CYB5R2

GPX3:

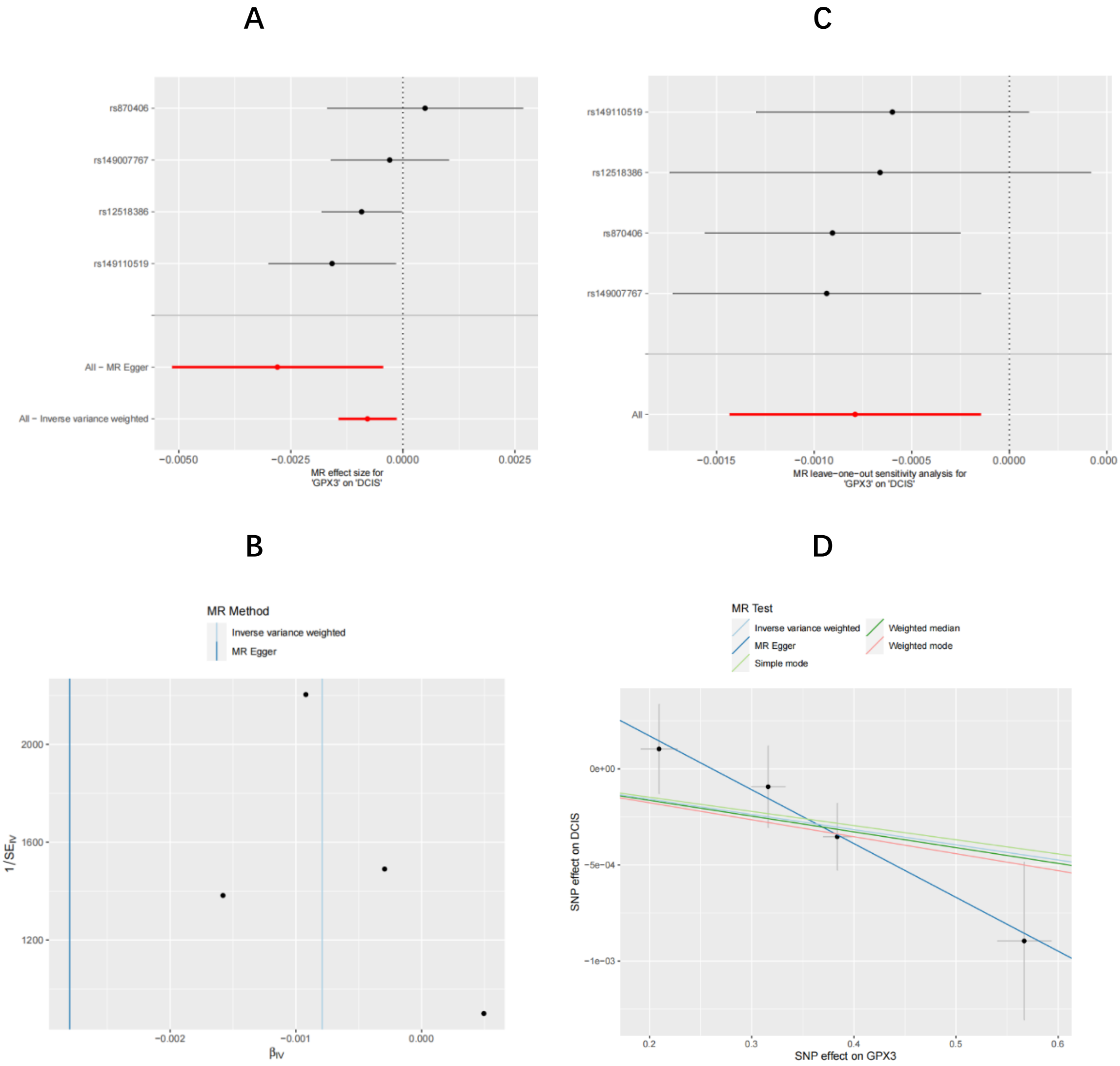

A
rs870406
rs149007767
rs12518386
rs149110519
All − MR Egger
All − Inverse variance weighted
−0.0050
−0.0025
0.0000
0.0025
MR effect size for 'GPX3' on 'DCIS'
C
rs149110519
rs12518386
rs870406
rs149007767
All
−0.0015
−0.0010
−0.0005
0.0000
0.000
MR leave−one−out sensitivity analysis for 'GPX3' on 'DCIS'
B
MR Method
Inverse variance weighted
MR Egger
2000
1600
1200
$1/SE_{IV}$
−0.002
−0.001
0.000
β_{IV}
D
MR Test
Inverse variance weighted
MR Egger
Simple mode
Weighted median
Weighted mode
0e+00
−5e−04
−1e−03
SNP effect on DCIS
0.2
0.3
0.4
0.5
0.6
SNP effect on GPX3

KLHDC4:

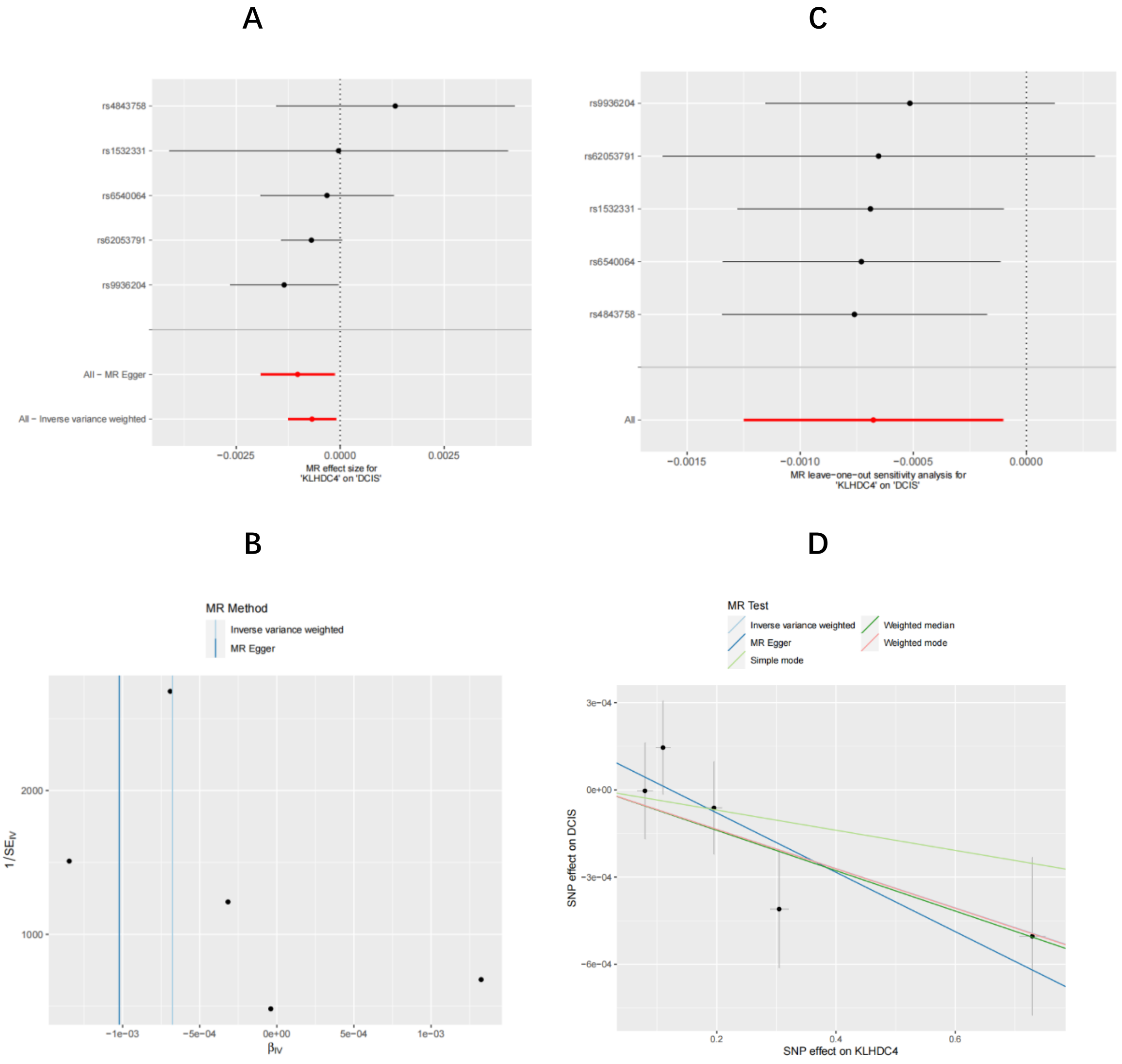
A
rs4843758
rs1532331
rs6540064
rs62053791
rs9936204
All − MR Egger
All − Inverse variance weighted
−0.0025
0.0000
0.0025
MR effect size for
'KLHDC4' on 'DCIS'
C
rs9936204
rs62053791
rs1532331
rs6540064
rs4843758
All
−0.0015
−0.0010
−0.0005
0.0000
MR leave−one−out sensitivity analysis for
'KLHDC4' on 'DCIS'
B
MR Method
Inverse variance weighted
MR Egger
2000
1000
1/SE_IV
−1e−03
−5e−04
0e+00
5e−04
1e−03
β_IV
D
MR Test
Inverse variance weighted
MR Egger
Simple mode
Weighted median
Weighted mode
3e−04
0e+00
−3e−04
−6e−04
SNP effect on DCIS
0.2
0.4
0.6
SNP effect on KLHDC4

LGALS8:

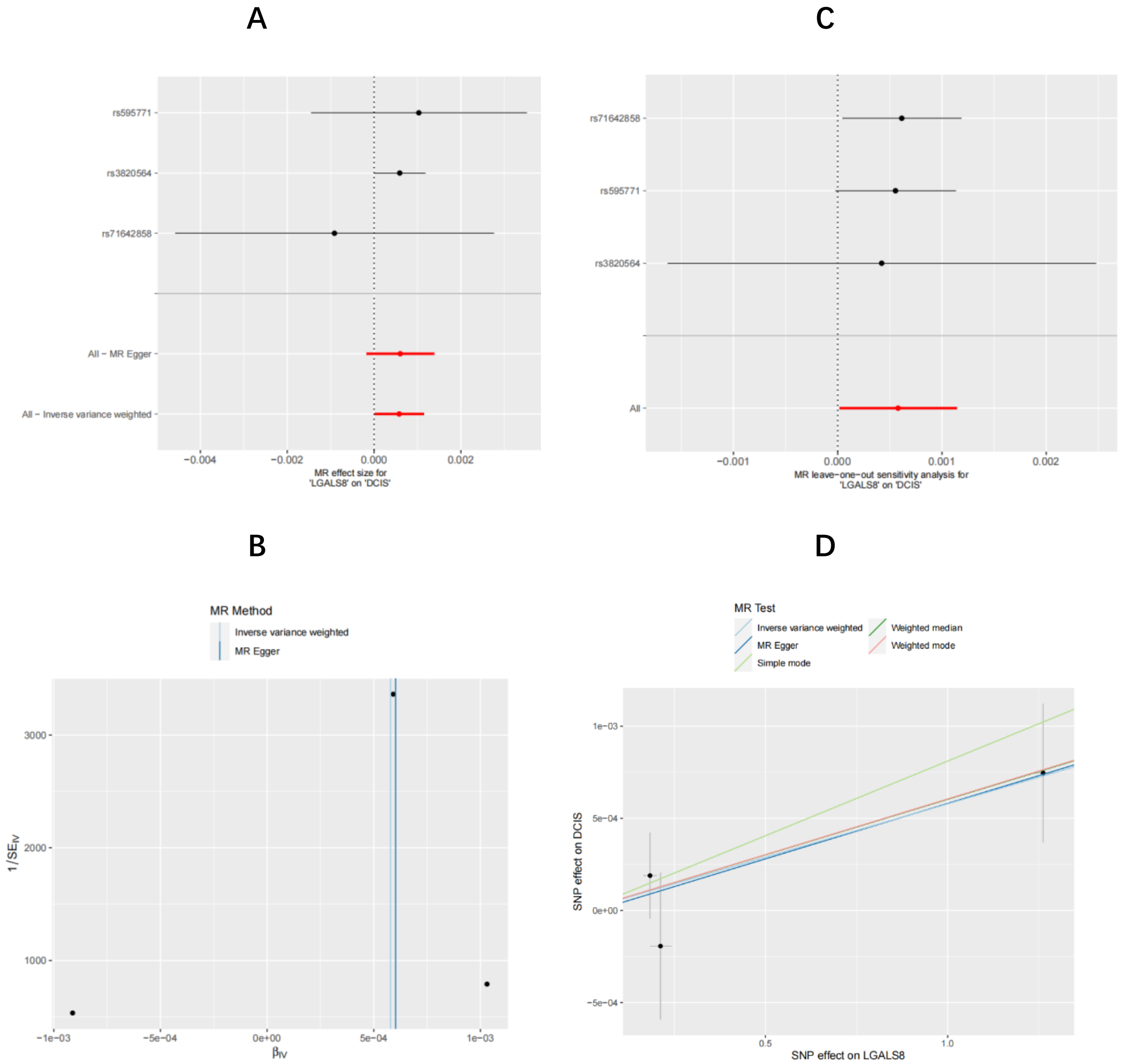
A
rs595771
rs3820564
rs71642858
All − MR Egger
All − Inverse variance weighted
−0.004
−0.002
0.000
0.002
MR effect size for
'LGALS8' on 'DCIS'
C
rs71642858
rs595771
rs3820564
All
−0.001
0.000
0.001
0.002
MR leave−one−out sensitivity analysis for
'LGALS8' on 'DCIS'
B
MR Method
Inverse variance weighted
MR Egger
3000
2000
1000
1/SE$_{IV}$
−1e−03
−5e−04
0e+00
5e−04
1e−03
β_{IV}
D
MR Test
Inverse variance weighted
MR Egger
Simple mode
Weighted median
Weighted mode
1e−03
5e−04
0e+00
−5e−04
SNP effect on DCIS
0.5
1.0
SNP effect on LGALS8

MAPKAPK3:

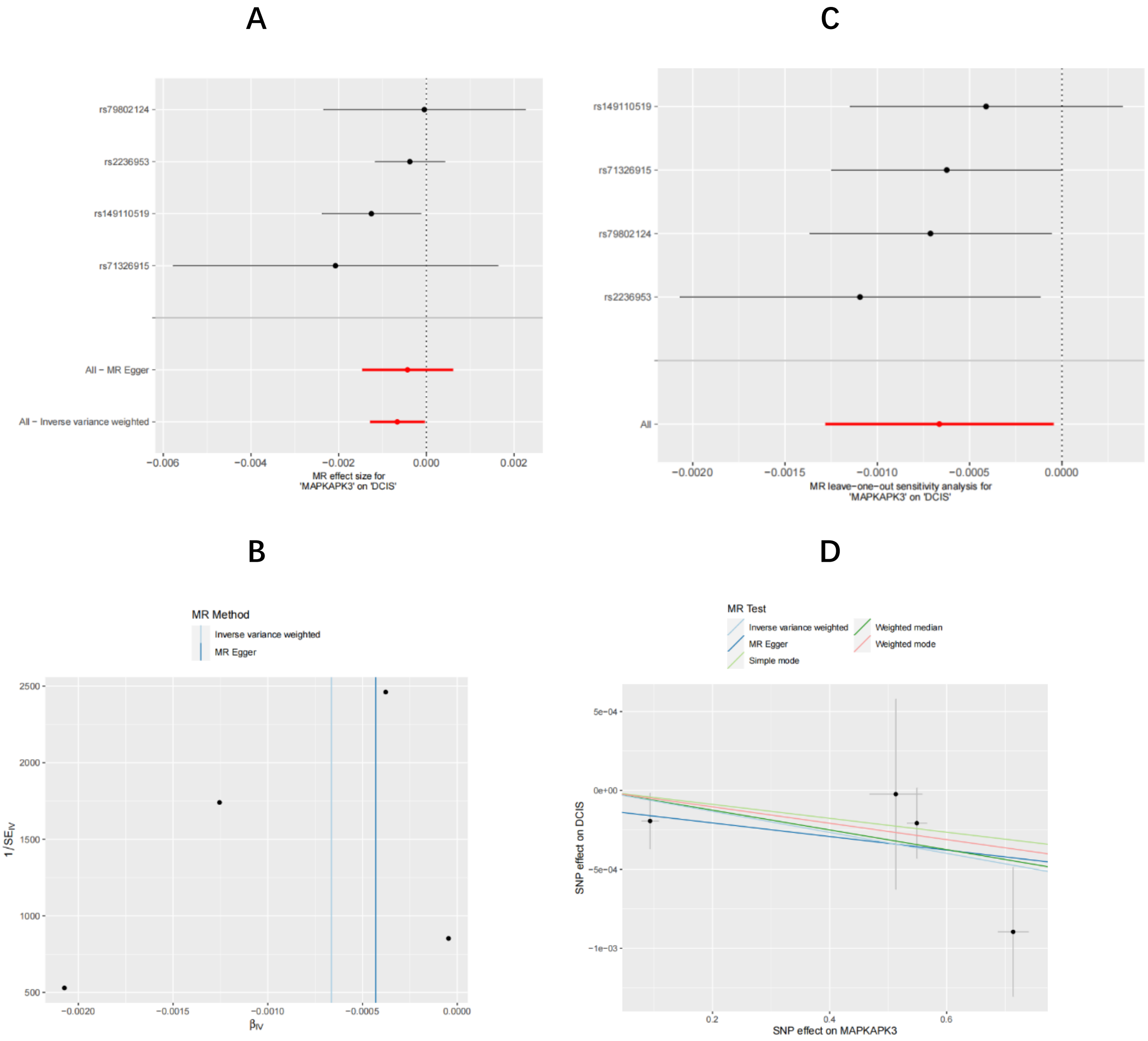
A
rs79802124
rs2236953
rs149110519
rs71326915
All − MR Egger
All − Inverse variance weighted
−0.006
−0.004
−0.002
0.000
0.002
MR effect size for
'MAPKAPK3' on 'DCIS'
C
rs149110519
rs71326915
rs79802124
rs2236953
All
−0.0020
−0.0015
−0.0010
−0.0005
0.0000
MR leave−one−out sensitivity analysis for
'MAPKAPK3' on 'DCIS'
B
MR Method
Inverse variance weighted
MR Egger
2500
2000
1500
1000
500
1/SE_IV
−0.0020
−0.0015
−0.0010
−0.0005
0.0000
β_IV
D
MR Test
Inverse variance weighted
MR Egger
Simple mode
Weighted median
Weighted mode
5e−04
0e+00
−5e−04
−1e−03
SNP effect on DCIS
0.2
0.4
0.6
SNP effect on MAPKAPK3

PTPN12:

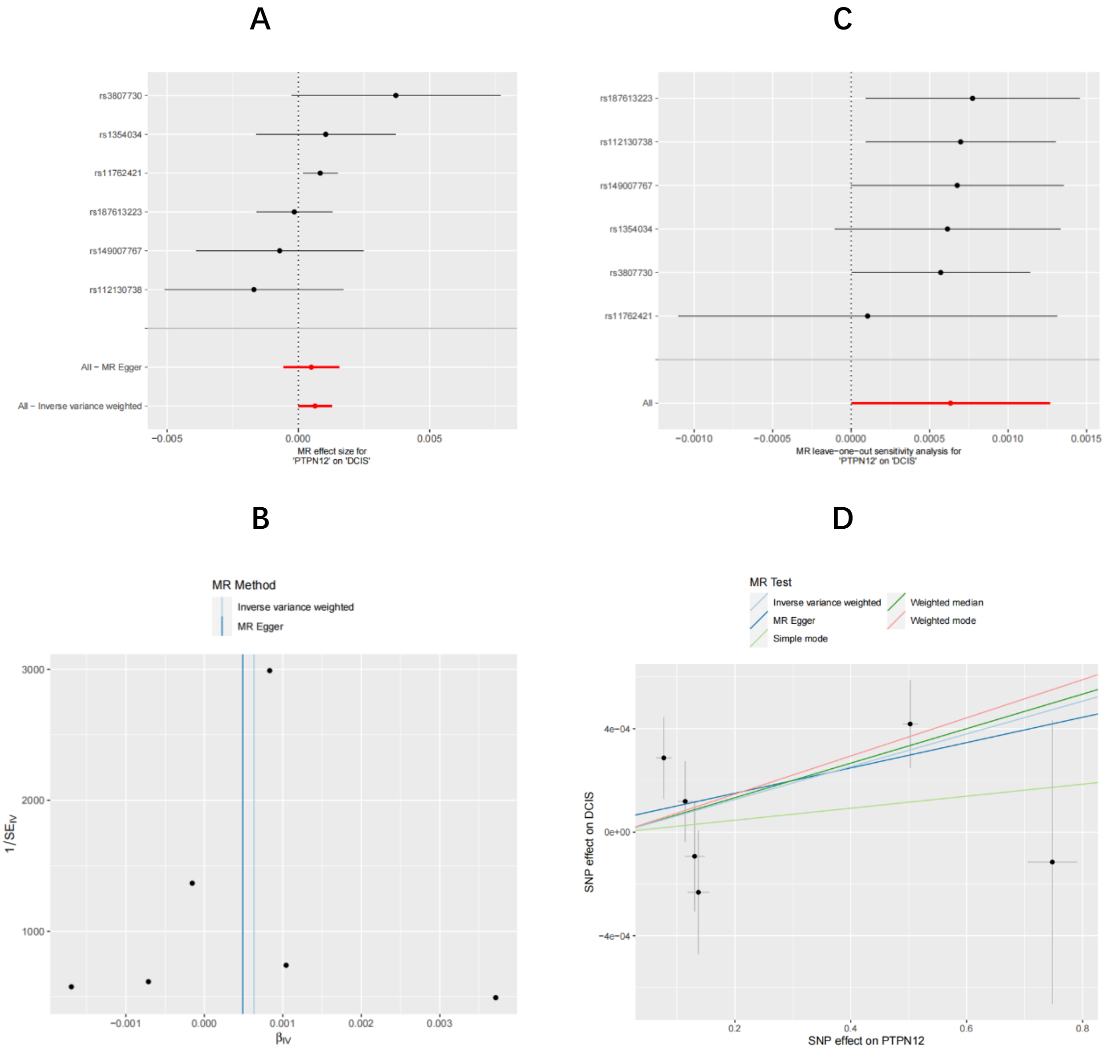
A
C
B
D
rs3807730
rs1354034
rs11762421
rs187613223
rs149007767
rs112130738
All – MR Egger
All – Inverse variance weighted
MR effect size for
'PTPN12' on 'DCIS'
All
MR leave–one–out sensitivity analysis for
'PTPN12' on 'DCIS'
MR Method
Inverse variance weighted
MR Egger
1/SE_IV
β_IV
MR Test
Inverse variance weighted
MR Egger
Simple mode
Weighted median
Weighted mode
SNP effect on DCIS
SNP effect on PTPN12

RAB3IL1:

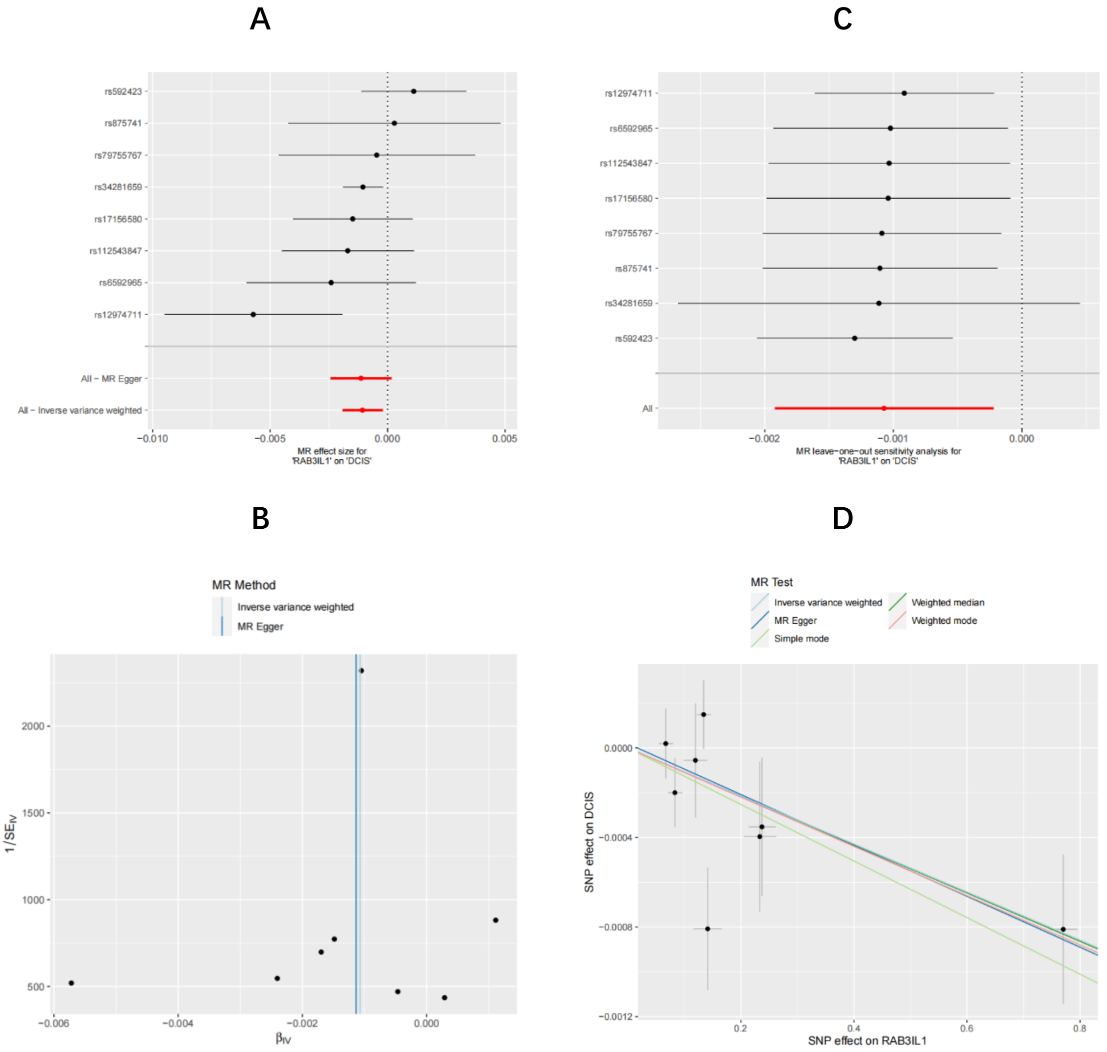
A
rs592423
rs875741
rs79755767
rs34281659
rs17156580
rs112543847
rs6592965
rs12974711
All − MR Egger
All − Inverse variance weighted
−0.010
−0.005
0.000
0.005
MR effect size for
'RAB3IL1' on 'DCIS'
C
rs12974711
rs6592965
rs112543847
rs17156580
rs79755767
rs875741
rs34281659
rs592423
All
−0.002
−0.001
0.000
MR leave−one−out sensitivity analysis for
'RAB3IL1' on 'DCIS'
B
MR Method
Inverse variance weighted
MR Egger
2000
1500
1000
500
1/SE_IV
−0.006
−0.004
−0.002
0.000
β_IV
D
MR Test
Inverse variance weighted
MR Egger
Simple mode
Weighted median
Weighted mode
0.0000
−0.0004
−0.0008
−0.0012
SNP effect on DCIS
0.2
0.4
0.6
0.8
SNP effect on RAB3IL1

RASA3:

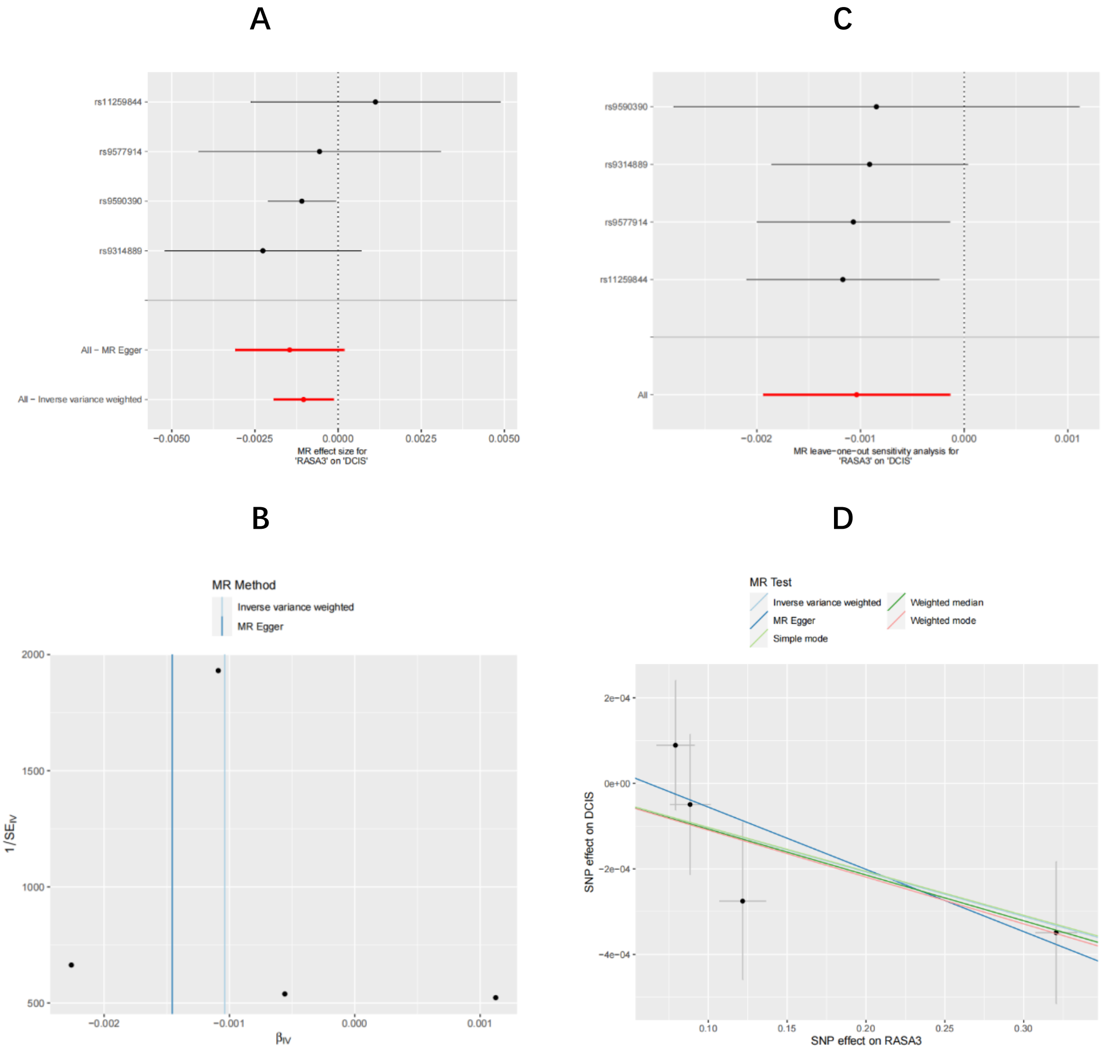

A
rs11259844
rs9577914
rs9590390
rs9314889
All − MR Egger
All − Inverse variance weighted
−0.0050
−0.0025
0.0000
0.0025
0.0050
MR effect size for
'RASA3' on 'DCIS'
C
rs9590390
rs9314889
rs9577914
rs11259844
All
−0.002
−0.001
0.000
0.001
MR leave−one−out sensitivity analysis for
'RASA3' on 'DCIS'
B
MR Method
Inverse variance weighted
MR Egger
2000
1500
1000
500
1/SE_IV
−0.002
−0.001
0.000
0.001
β_IV
D
MR Test
Inverse variance weighted
MR Egger
Simple mode
Weighted median
Weighted mode
2e−04
0e+00
−2e−04
−4e−04
SNP effect on DCIS
0.10
0.15
0.20
0.25
0.30
SNP effect on RASA3

RNGTT:

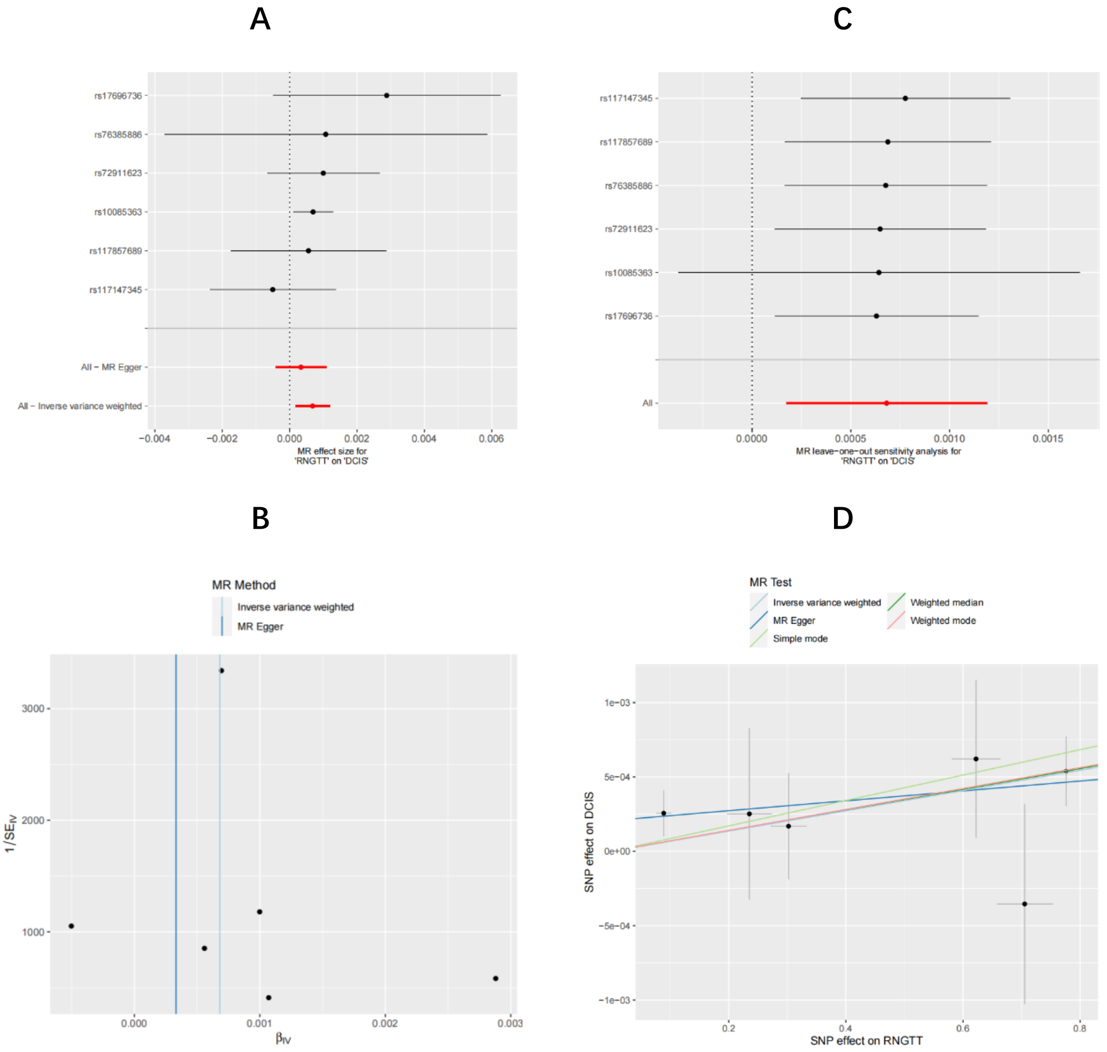
A
rs17696736
rs76385886
rs72911623
rs10085363
rs117857689
rs117147345
All − MR Egger
All − Inverse variance weighted
−0.004
−0.002
0.000
0.002
0.004
0.006
MR effect size for
'RNGTT' on 'DCIS'
C
rs117147345
rs117857689
rs76385886
rs72911623
rs10085363
rs17696736
All
0.0000
0.0005
0.0010
0.0015
MR leave−one−out sensitivity analysis for
'RNGTT' on 'DCIS'
B
MR Method
Inverse variance weighted
MR Egger
3000
2000
1000
$1/SE_{IV}$
0.000
0.001
0.002
0.003
β_{IV}
D
MR Test
Inverse variance weighted
MR Egger
Simple mode
Weighted median
Weighted mode
1e−03
5e−04
0e+00
−5e−04
−1e−03
SNP effect on DCIS
0.2
0.4
0.6
0.8
SNP effect on RNGTT

TSPAN4:

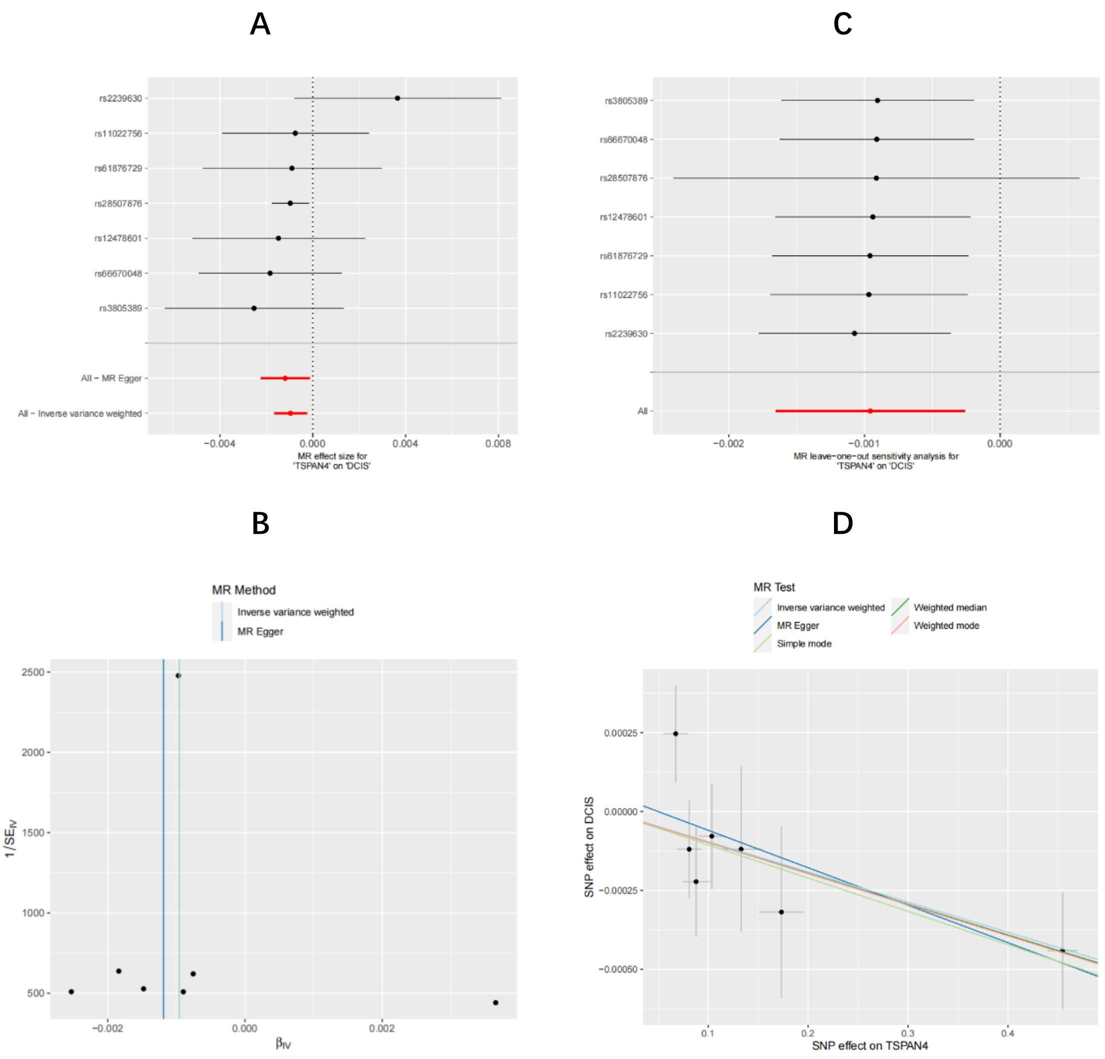
A
rs2239630
rs11022756
rs61876729
rs28507876
rs12478601
rs66670048
rs3805389
All − MR Egger
All − Inverse variance weighted
−0.004
0.000
0.004
0.008
MR effect size for
'TSPAN4' on 'DCIS'
C
rs3805389
rs66670048
rs28507876
rs12478601
rs61876729
rs11022756
rs2239630
All
−0.002
−0.001
0.000
MR leave−one−out sensitivity analysis for
'TSPAN4' on 'DCIS'
B
MR Method
Inverse variance weighted
MR Egger
2500
2000
1500
1000
500
1/SE_IV
−0.002
0.000
0.002
β_IV
D
MR Test
Inverse variance weighted
MR Egger
Simple mode
Weighted median
Weighted mode
0.00025
0.00000
−0.00025
−0.00050
SNP effect on DCIS
0.1
0.2
0.3
0.4
SNP effect on TSPAN4

YTHDC2:

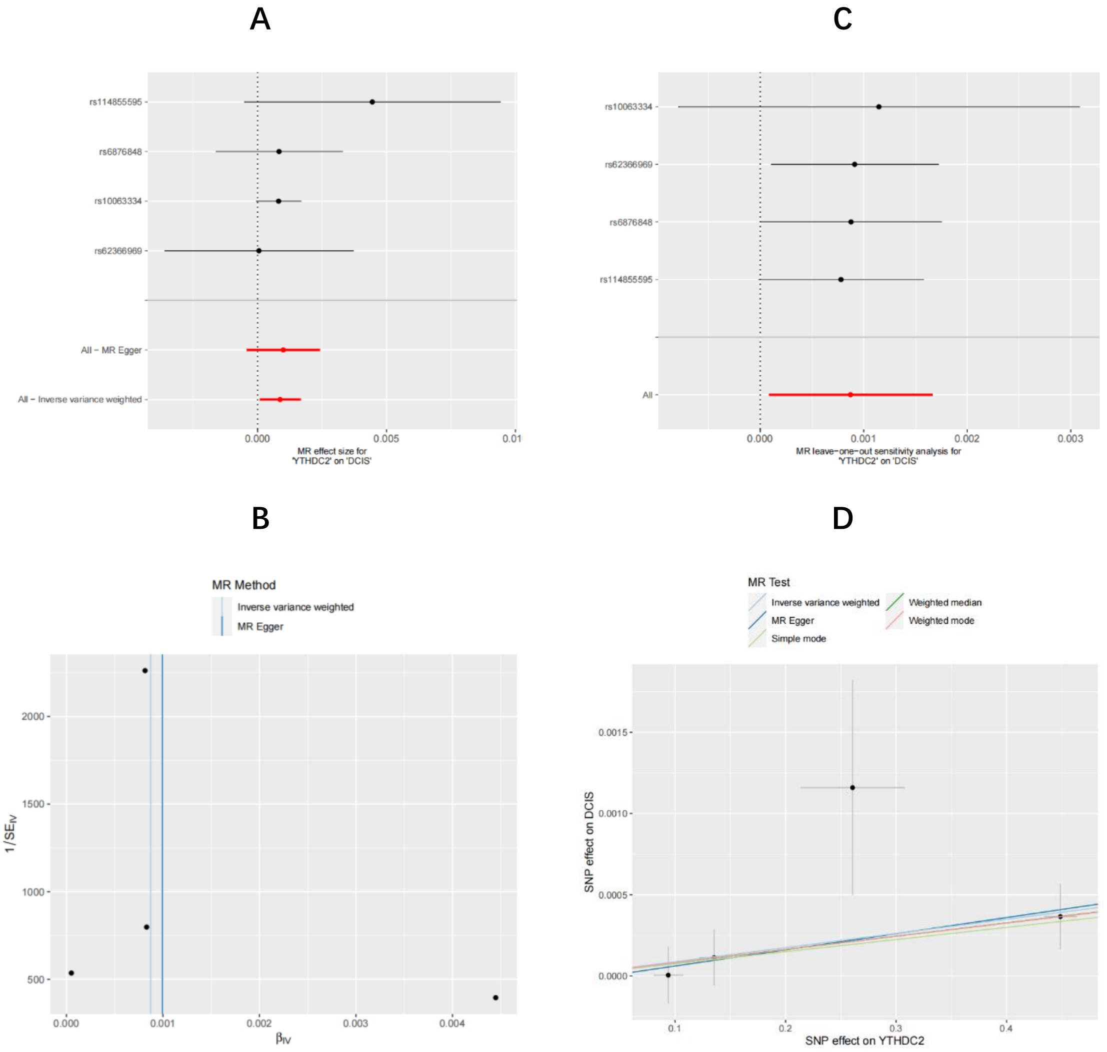
A
C
rs114855595
rs6876848
rs10063334
rs62366969
All - MR Egger
All - Inverse variance weighted
0.000
0.005
0.01
MR effect size for
'YTHDC2' on 'DCIS'
rs10063334
rs62366969
rs6876848
rs114855595
All
0.000
0.001
0.002
0.003
MR leave-one-out sensitivity analysis for
'YTHDC2' on 'DCIS'
B
D
MR Method
Inverse variance weighted
MR Egger
2000
1500
1000
500
1/SE$_{IV}$
0.000
0.001
0.002
0.003
0.004
β$_{IV}$
MR Test
Inverse variance weighted
MR Egger
Simple mode
Weighted median
Weighted mode
0.0015
0.0010
0.0005
0.0000
SNP effect on DCIS
0.1
0.2
0.3
0.4
SNP effect on YTHDC2

ZFP37:

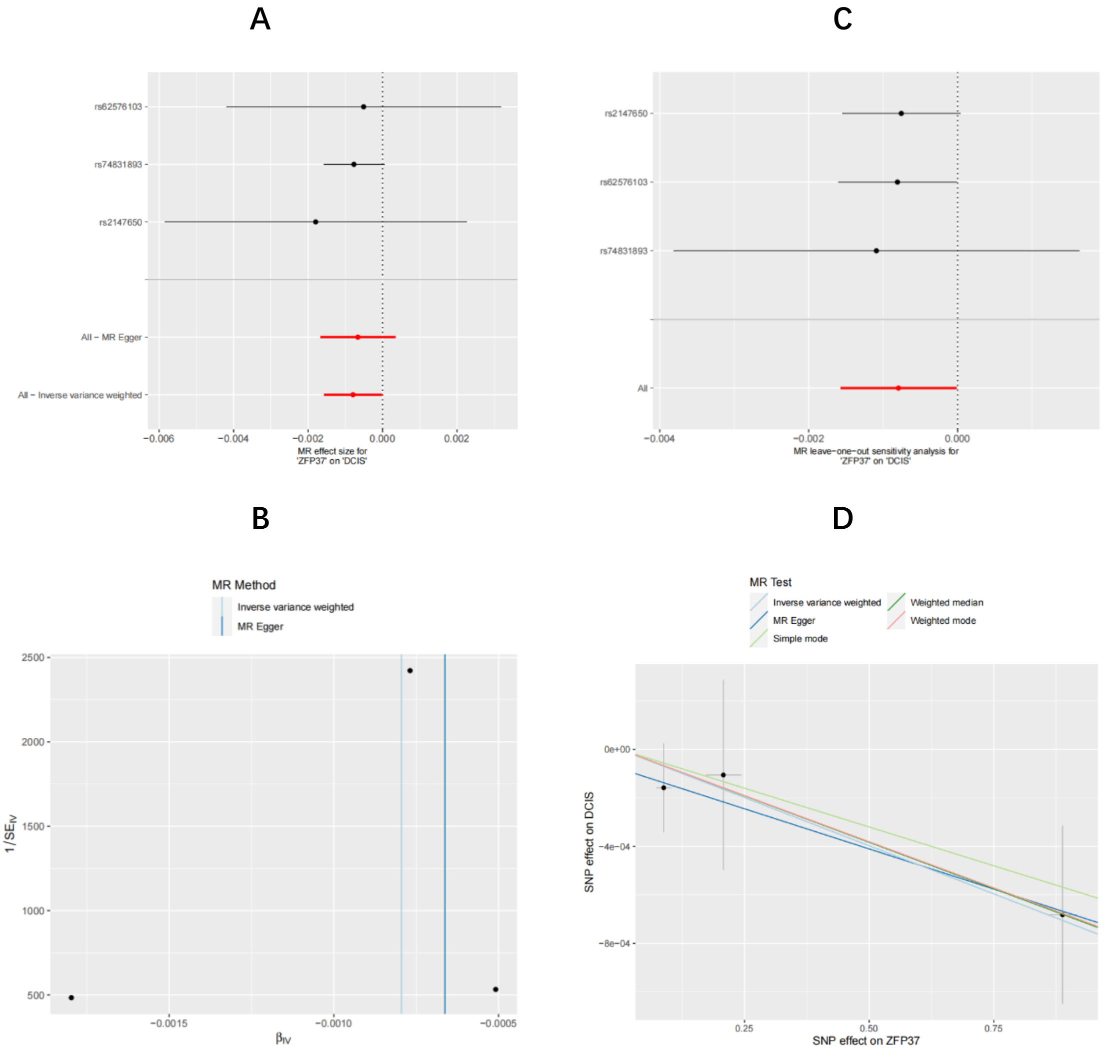

A
rs62576103
rs74831893
rs2147650
All − MR Egger
All − Inverse variance weighted
−0.006
−0.004
−0.002
0.000
0.002
MR effect size for
'ZFP37' on 'DCIS'
C
rs2147650
rs62576103
rs74831893
All
−0.004
−0.002
0.000
MR leave−one−out sensitivity analysis for
'ZFP37' on 'DCIS'
B
MR Method
Inverse variance weighted
MR Egger
2500
2000
1500
1000
500
1/SE_IV
−0.0015
−0.0010
−0.0005
β_IV
D
MR Test
Inverse variance weighted
MR Egger
Simple mode
Weighted median
Weighted mode
0e+00
−4e−04
−8e−04
SNP effect on DCIS
0.25
0.50
0.75
SNP effect on ZFP37
