# Supplementary material for: Integration of eQTL and GEO Datasets to Identify Genes Associated with Breast Ductal Carcinoma In Situ
Source: Curr Issues Mol Biol. 2025 Sep 11;47(9):747. doi: 10.3390/cimb47090747 (PMC12468441; doi:10.3390/cimb47090747)
Supplement: Supplementary file 1 [file cimb-47-00747-s001.zip › Supplementary Figures S2.pdf]

Supplemental Figures S2. Details of GSEA analysis results of some DEGs related to immune cell infiltration. Each line in different colors represents a distinct enrichment pathway. In this figure, we have listed a total of five of the most significant types of pathways. The upper part of the figure specifically presents the results of the GSEA GO enrichment analysis, while the lower part separately shows the outcomes of the GSEA KEGG enrichment analysis.

APOBEC3G

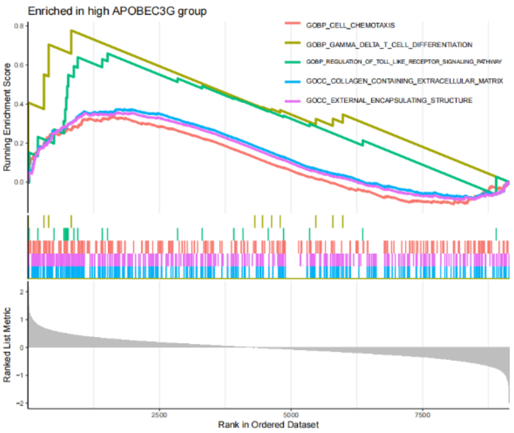

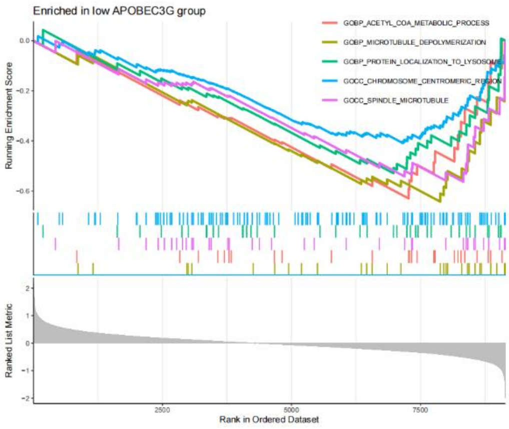

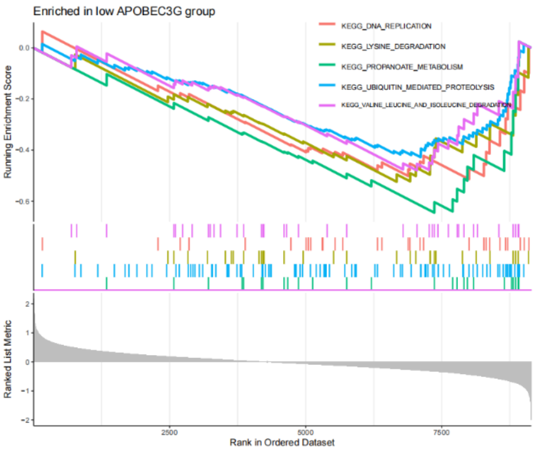

CYB5R2

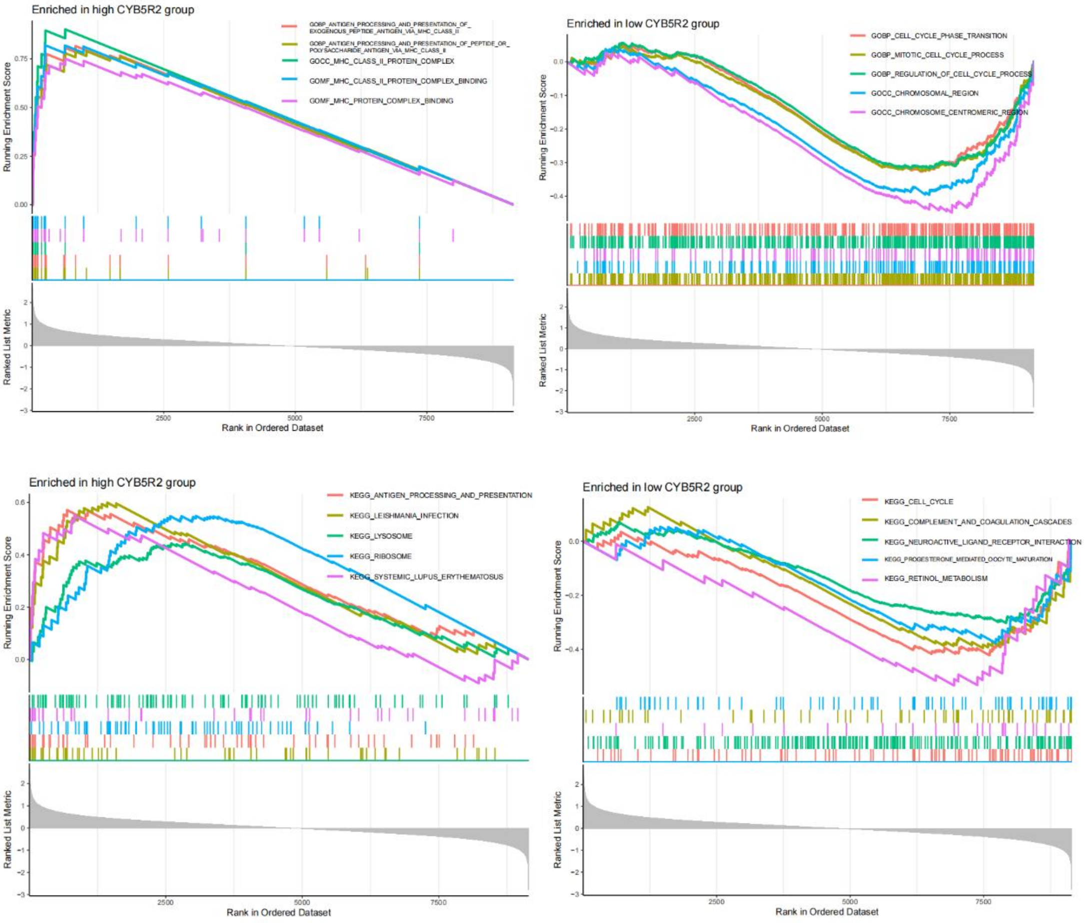
Enriched in high CYB5R2 group
GOBP_ANTIGEN_PROCESSING_AND_PRESENTATION_OF_EXOGENOUS_PEPTIDE_ANTIGEN_VIA_MHC_CLASS_II
GOBP_ANTIGEN_PROCESSING_AND_PRESENTATION_OF_PEPTIDE_OR_POLYSACCHARIDE_ANTIGEN_VIA_MHC_CLASS_II
GOCC_MHC_CLASS_II_PROTEIN_COMPLEX
GOMF_MHC_CLASS_II_PROTEIN_COMPLEX_BINDING
GOMF_MHC_PROTEIN_COMPLEX_BINDING
Running Enrichment Score
Ranked List Metric
Rank in Ordered Dataset
Enriched in low CYB5R2 group
GOBP_CELL_CYCLE_PHASE_TRANSITION
GOBP_MITOTIC_CELL_CYCLE_PROCESS
GOBP_REGULATION_OF_CELL_CYCLE_PROCESS
GOCC_CHROMOSOMAL_REGION
GOCC_CHROMOSOME_CENTROMERIC_REGION
Running Enrichment Score
Ranked List Metric
Rank in Ordered Dataset
Enriched in high CYB5R2 group
KEGG_ANTIGEN_PROCESSING_AND_PRESENTATION
KEGG_LEISHMANIA_INFECTION
KEGG_LYSOSOME
KEGG_RIBOSOME
KEGG_SYSTEMIC_LUPUS_ERYTHEMATOSUS
Running Enrichment Score
Ranked List Metric
Rank in Ordered Dataset
Enriched in low CYB5R2 group
KEGG_CELL_CYCLE
KEGG_COMPLEMENT_AND_COAGULATION_CASCADES
KEGG_NEUROACTIVE_LIGAND_RECEPTOR_INTERACTION
KEGG_PROGESTERONE_MEDIATED_OOCYTE_MATURATION
KEGG_RETINOL_METABOLISM
Running Enrichment Score
Ranked List Metric
Rank in Ordered Dataset

GPX3

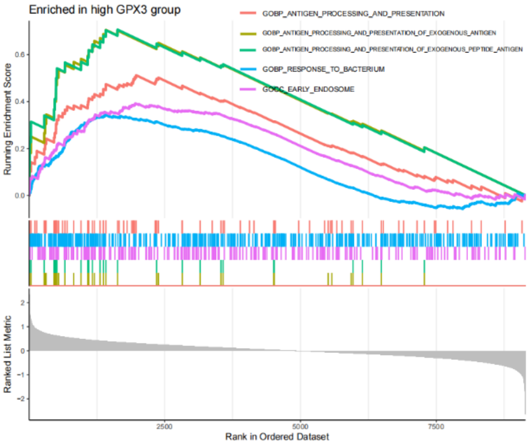
Enriched in high GPX3 group
GOBP_ANTIGEN_PROCESSING_AND_PRESENTATION
GOBP_ANTIGEN_PROCESSING_AND_PRESENTATION_OF_EXOGENOUS_ANTIGEN
GOBP_ANTIGEN_PROCESSING_AND_PRESENTATION_OF_EXOGENOUS_PEPTIDE_ANTIGEN
GOBP_RESPONSE_TO_BACTERIUM
GOCC_EARLY_ENDOSOME
Running Enrichment Score
Ranked List Metric
Rank in Ordered Dataset

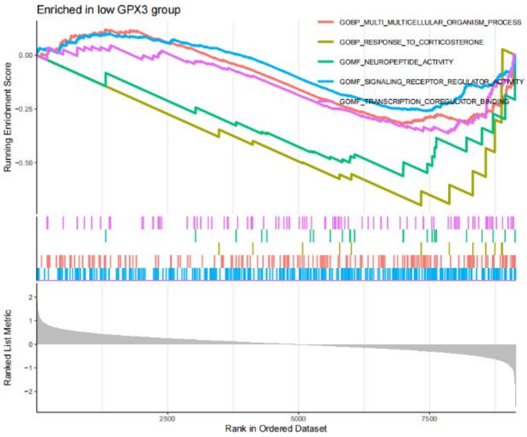
Enriched in low GPX3 group
GOBP_MULTI_MULTICELLULAR_ORGANISM_PROCESS
GOBP_RESPONSE_TO_CORTICOSTERONE
GOMF_NEUROPEPTIDE_ACTIVITY
GOMF_SIGNALING_RECEPTOR_REGULATOR_ACTIVITY
GOMF_TRANSCRIPTION_COREGULATOR_BINDING
Running Enrichment Score
Ranked List Metric
Rank in Ordered Dataset

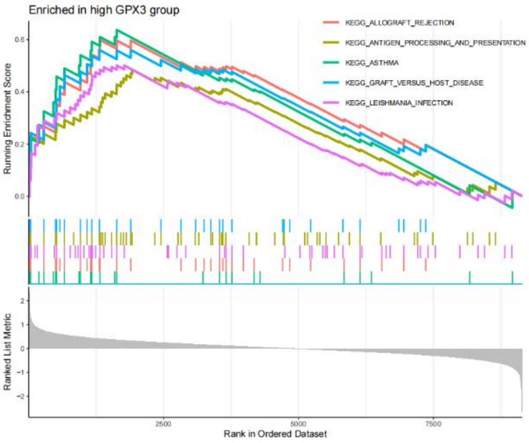
Enriched in high GPX3 group
KEGG_ALLOGRAFT_REJECTION
KEGG_ANTIGEN_PROCESSING_AND_PRESENTATION
KEGG_ASTHMA
KEGG_GRAFT_VERSUS_HOST_DISEASE
KEGG_LEISHMANIA_INFECTION
Running Enrichment Score
Ranked List Metric
Rank in Ordered Dataset

KLHDC4

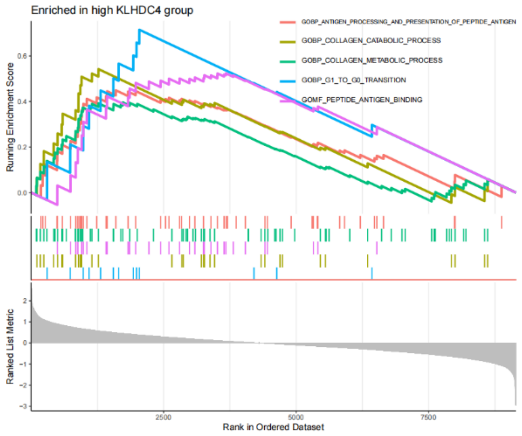

Enriched in high KLHDC4 group
GOBP_ANTIGEN_PROCESSING_AND_PRESENTATION_OF_PEPTIDE_ANTIGEN
GOBP_COLLAGEN_CATABOLIC_PROCESS
GOBP_COLLAGEN_METABOLIC_PROCESS
GOBP_G1_TO_G0_TRANSITION
GOMF_PEPTIDE_ANTIGEN_BINDING
Running Enrichment Score
Ranked List Metric
Rank in Ordered Dataset

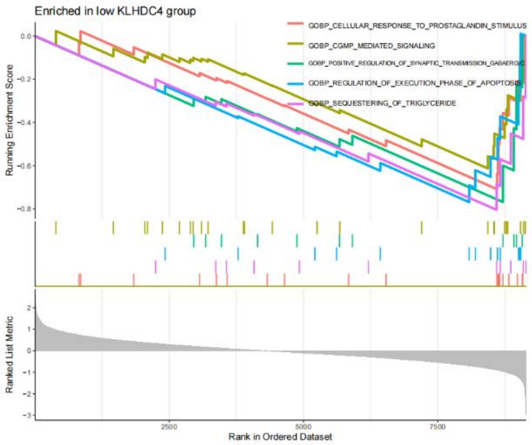

Enriched in low KLHDC4 group
GOBP_CELLULAR_RESPONSE_TO_PROSTAGLANDIN_STIMULUS
GOBP_CGMP_MEDIATED_SIGNALING
GOBP_POSITIVE_REGULATION_OF_SYNAPTIC_TRANSMISSION_GABAERGIC
GOBP_REGULATION_OF_EXECUTION_PHASE_OF_APOPTOSIS
GOBP_SEQUESTERING_OF_TRIGLYCERIDE
Running Enrichment Score
Ranked List Metric
Rank in Ordered Dataset

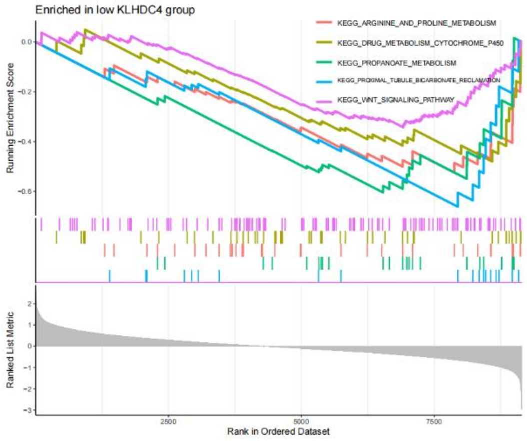

Enriched in low KLHDC4 group
KEGG_ARGININE_AND_PROLINE_METABOLISM
KEGG_DRUG_METABOLISM_CYTOCHROME_P450
KEGG_PROPANOATE_METABOLISM
KEGG_PROXIMAL_TUBULE_BICARBONATE_RECLAMATION
KEGG_WNT_SIGNALING_PATHWAY
Running Enrichment Score
Ranked List Metric
Rank in Ordered Dataset

LGALS8

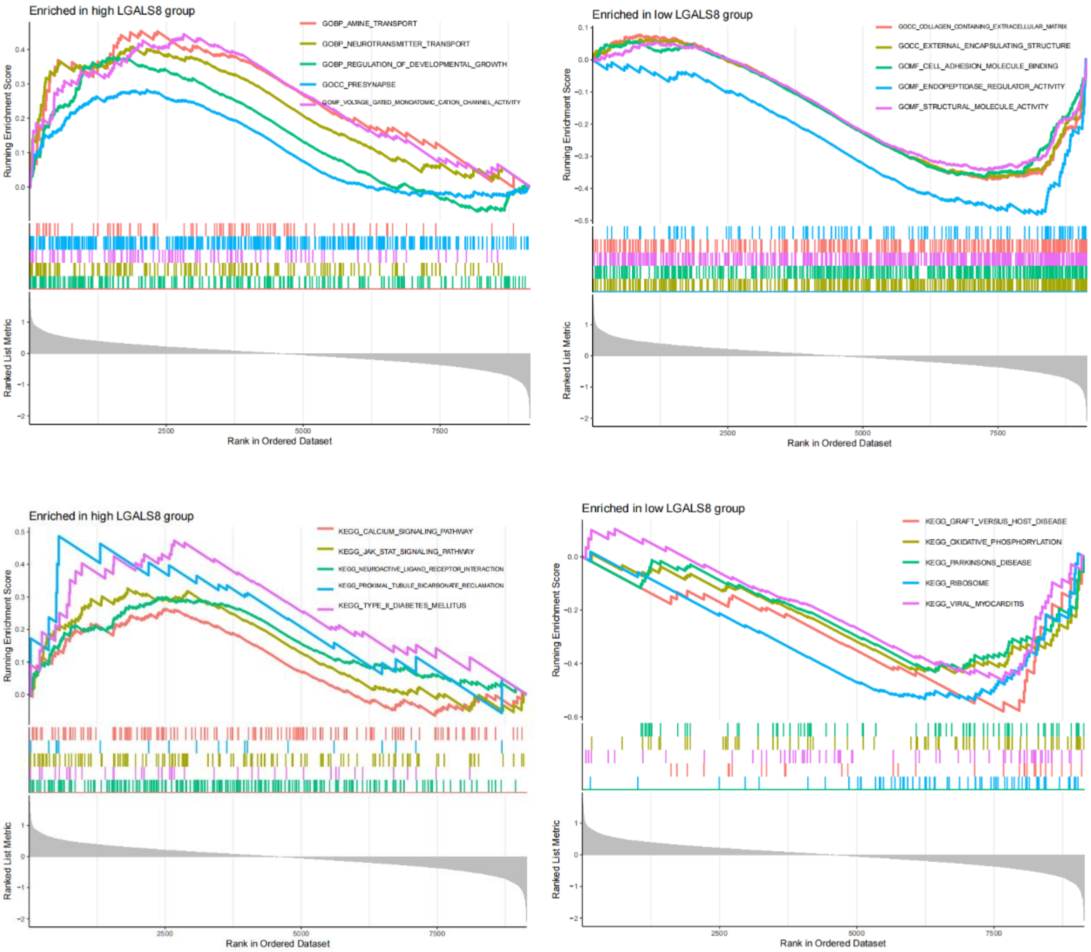
Enriched in high LGALS8 group
GOBP_AMINE_TRANSPORT
GOBP_NEUROTRANSMITTER_TRANSPORT
GOBP_REGULATION_OF_DEVELOPMENTAL_GROWTH
GOCC_PRESYNAPSE
GOMF_VOLTAGE_GATED_MONOATOMIC_CATION_CHANNEL_ACTIVITY
Running Enrichment Score
Ranked List Metric
Rank in Ordered Dataset
Enriched in low LGALS8 group
GOCC_COLLAGEN_CONTAINING_EXTRACELLULAR_MATRIX
GOCC_EXTERNAL_ENCAPSULATING_STRUCTURE
GOMF_CELL_ADHESION_MOLECULE_BINDING
GOMF_ENDOPEPTIDASE_REGULATOR_ACTIVITY
GOMF_STRUCTURAL_MOLECULE_ACTIVITY
Running Enrichment Score
Ranked List Metric
Rank in Ordered Dataset
Enriched in high LGALS8 group
KEGG_CALCIUM_SIGNALING_PATHWAY
KEGG_JAK_STAT_SIGNALING_PATHWAY
KEGG_NEUROACTIVE_LIGAND_RECEPTOR_INTERACTION
KEGG_PROXIMAL_TUBULE_BICARBONATE_RECLAMATION
KEGG_TYPE_II_DIABETES_MELLITUS
Running Enrichment Score
Ranked List Metric
Rank in Ordered Dataset
Enriched in low LGALS8 group
KEGG_GRAFT_VERSUS_HOST_DISEASE
KEGG_OXIDATIVE_PHOSPHORYLATION
KEGG_PARKINSONS_DISEASE
KEGG_RIBOSOME
KEGG_VIRAL_MYOCARDITIS
Running Enrichment Score
Ranked List Metric
Rank in Ordered Dataset

MAPKAPK3

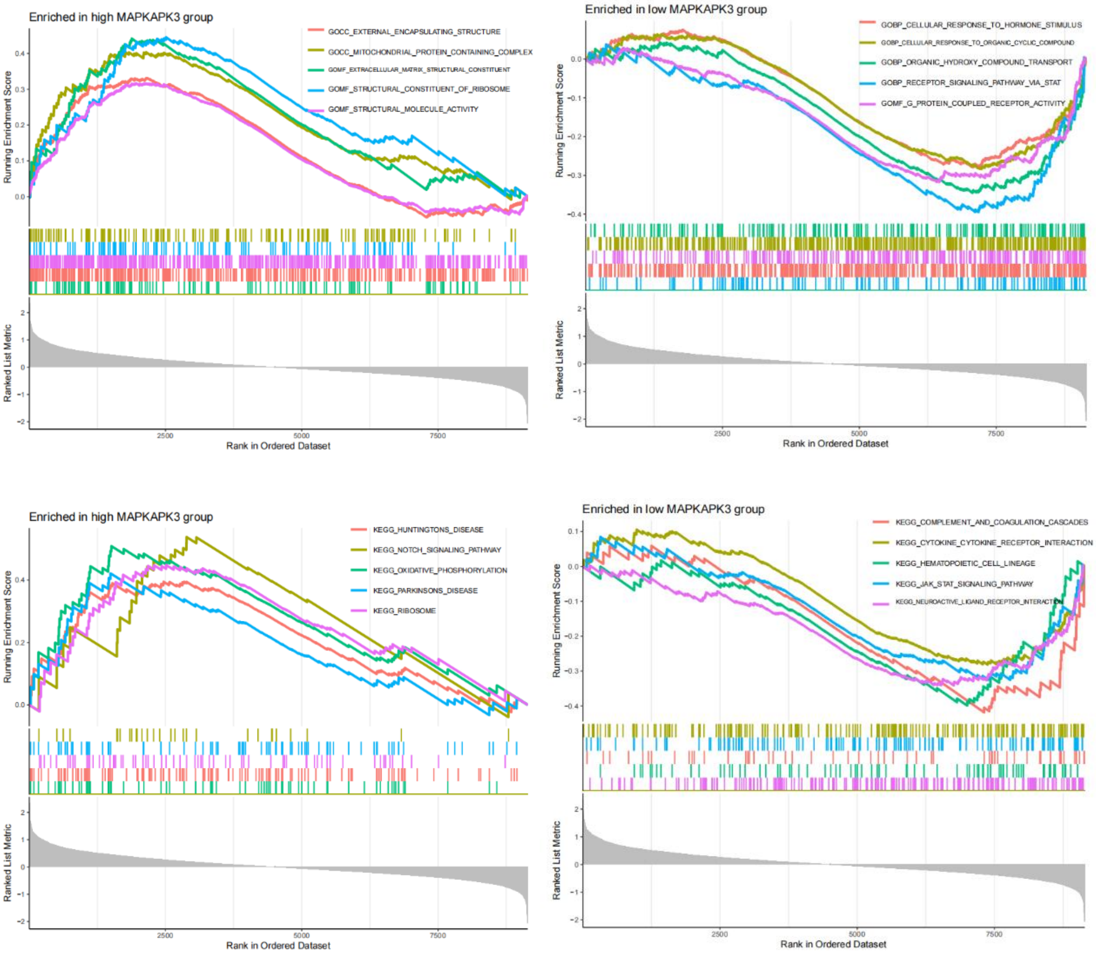
Enriched in high MAPKAPK3 group
GOCC_EXTERNAL_ENCAPSULATING_STRUCTURE
GOCC_MITOCHONDRIAL_PROTEIN_CONTAINING_COMPLEX
GOMF_EXTRACELLULAR_MATRIX_STRUCTURAL_CONSTITUENT
GOMF_STRUCTURAL_CONSTITUENT_OF_RIBOSOME
GOMF_STRUCTURAL_MOLECULE_ACTIVITY
Running Enrichment Score
Ranked List Metric
Rank in Ordered Dataset
Enriched in low MAPKAPK3 group
GOBP_CELLULAR_RESPONSE_TO_HORMONE_STIMULUS
GOBP_CELLULAR_RESPONSE_TO_ORGANIC_CYCLIC_COMPOUND
GOBP_ORGANIC_HYDROXY_COMPOUND_TRANSPORT
GOBP_RECEPTOR_SIGNALING_PATHWAY_VIA_STAT
GOMF_G_PROTEIN_COUPLED_RECEPTOR_ACTIVITY
Running Enrichment Score
Ranked List Metric
Rank in Ordered Dataset
Enriched in high MAPKAPK3 group
KEGG_HUNTINGTONS_DISEASE
KEGG_NOTCH_SIGNALING_PATHWAY
KEGG_OXIDATIVE_PHOSPHORYLATION
KEGG_PARKINSONS_DISEASE
KEGG_RIBOSOME
Running Enrichment Score
Ranked List Metric
Rank in Ordered Dataset
Enriched in low MAPKAPK3 group
KEGG_COMPLEMENT_AND_COAGULATION_CASCADES
KEGG_CYTOKINE_CYTOKINE_RECEPTOR_INTERACTION
KEGG_HEMATOPOIETIC_CELL_LINEAGE
KEGG_JAK_STAT_SIGNALING_PATHWAY
KEGG_NEUROACTIVE_LIGAND_RECEPTOR_INTERACTION
Running Enrichment Score
Ranked List Metric
Rank in Ordered Dataset

PTPN12

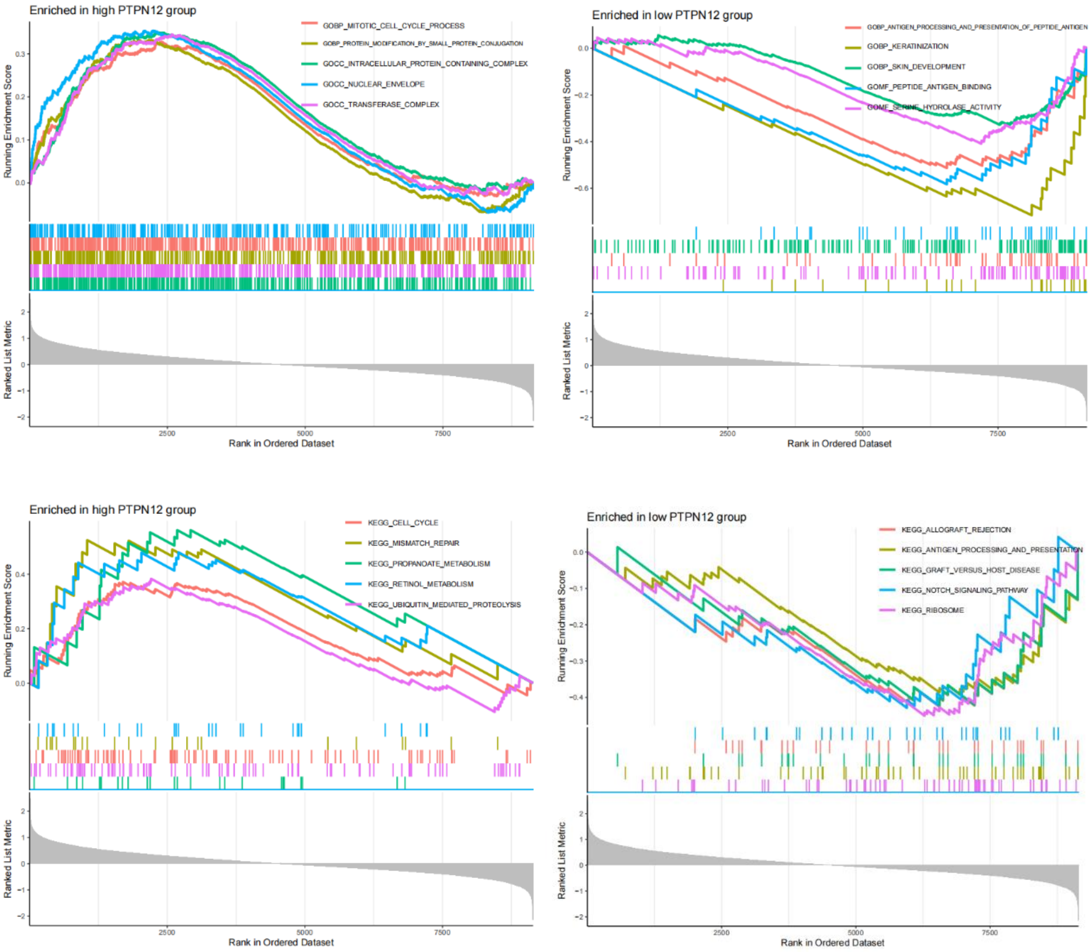
Enriched in high PTPN12 group
GOBP_MITOTIC_CELL_CYCLE_PROCESS
GOBP_PROTEIN_MODIFICATION_BY_SMALL_PROTEIN_CONJUGATION
GOCC_INTRACELLULAR_PROTEIN_CONTAINING_COMPLEX
GOCC_NUCLEAR_ENVELOPE
GOCC_TRANSFERASE_COMPLEX
Running Enrichment Score
Ranked List Metric
Rank in Ordered Dataset
Enriched in low PTPN12 group
GOBP_ANTIGEN_PROCESSING_AND_PRESENTATION_OF_PEPTIDE_ANTIGEN
GOBP_KERATINIZATION
GOBP_SKIN_DEVELOPMENT
GOMF_PEPTIDE_ANTIGEN_BINDING
GOMF_SERINE_HYDROLASE_ACTIVITY
Enriched in high PTPN12 group
KEGG_CELL_CYCLE
KEGG_MISMATCH_REPAIR
KEGG_PROPANOATE_METABOLISM
KEGG_RETINOL_METABOLISM
KEGG_UBIQUITIN_MEDIATED_PROTEOLYSIS
Enriched in low PTPN12 group
KEGG_ALLOGRAFT_REJECTION
KEGG_ANTIGEN_PROCESSING_AND_PRESENTATION
KEGG_GRAFT_VERSUS_HOST_DISEASE
KEGG_NOTCH_SIGNALING_PATHWAY
KEGG_RIBOSOME

RAB3IL1

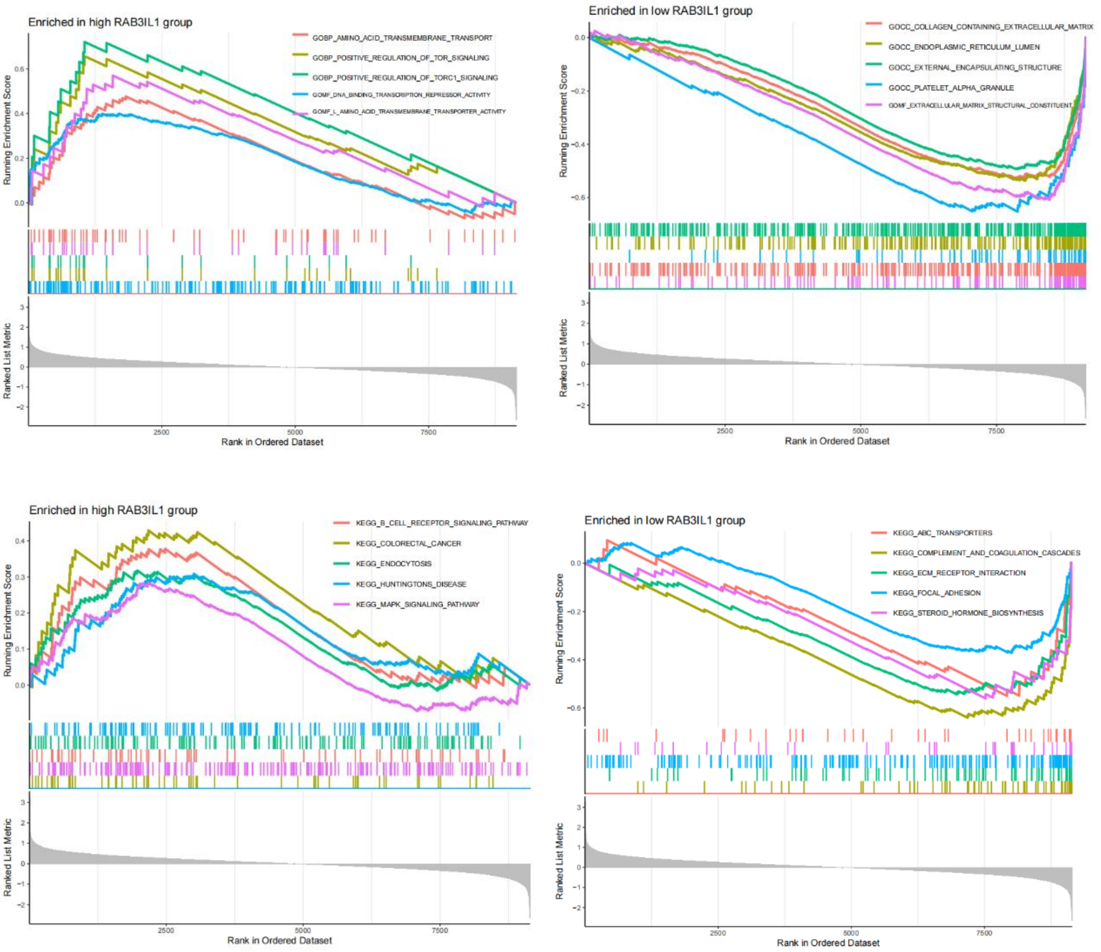

Enriched in high RAB3IL1 group
GOBP_AMINO_ACID_TRANSMEMBRANE_TRANSPORT
GOBP_POSITIVE_REGULATION_OF_TOR_SIGNALING
GOBP_POSITIVE_REGULATION_OF_TORC1_SIGNALING
GOMF_DNA_BINDING_TRANSCRIPTION_REPRESSOR_ACTIVITY
GOMF_L_AMINO_ACID_TRANSMEMBRANE_TRANSPORTER_ACTIVITY
Running Enrichment Score
Ranked List Metric
Rank in Ordered Dataset
Enriched in low RAB3IL1 group
GOCC_COLLAGEN_CONTAINING_EXTRACELLULAR_MATRIX
GOCC_ENDOPLASMIC_RETICULUM_LUMEN
GOCC_EXTERNAL_ENCAPSULATING_STRUCTURE
GOCC_PLATELET_ALPHA_GRANULE
GOMF_EXTRACELLULAR_MATRIX_STRUCTURAL_CONSTITUENT
Running Enrichment Score
Ranked List Metric
Rank in Ordered Dataset
Enriched in high RAB3IL1 group
KEGG_B_CELL_RECEPTOR_SIGNALING_PATHWAY
KEGG_COLORECTAL_CANCER
KEGG_ENDOCYTOSIS
KEGG_HUNTINGTONS_DISEASE
KEGG_MAPK_SIGNALING_PATHWAY
Running Enrichment Score
Ranked List Metric
Rank in Ordered Dataset
Enriched in low RAB3IL1 group
KEGG_ABC_TRANSPORTERS
KEGG_COMPLEMENT_AND_COAGULATION_CASCADES
KEGG_ECM_RECEPTOR_INTERACTION
KEGG_FOCAL_ADHESION
KEGG_STEROID_HORMONE_BIOSYNTHESIS
Running Enrichment Score
Ranked List Metric
Rank in Ordered Dataset

RASA3

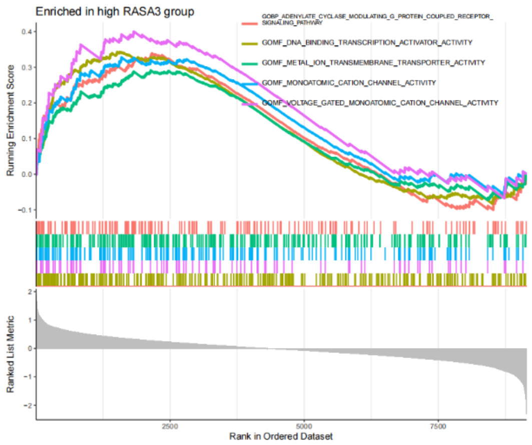

Enriched in high RASA3 group
GOBP_ADENYLATE_CYCLASE_MODULATING_G_PROTEIN_COUPLED_RECEPTOR_SIGNALING_PATHWAY
GOMF_DNA_BINDING_TRANSCRIPTION_ACTIVATOR_ACTIVITY
GOMF_METAL_ION_TRANSMEMBRANE_TRANSPORTER_ACTIVITY
GOMF_MONOATOMIC_CATION_CHANNEL_ACTIVITY
GOMF_VOLTAGE_GATED_MONOATOMIC_CATION_CHANNEL_ACTIVITY
Running Enrichment Score
Ranked List Metric
Rank in Ordered Dataset

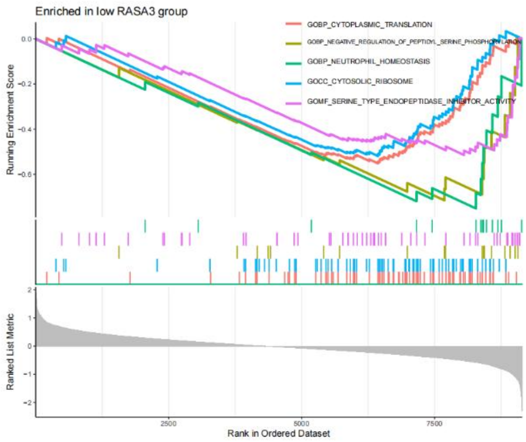

Enriched in low RASA3 group
GOBP_CYTOPLASMIC_TRANSLATION
GOBP_NEGATIVE_REGULATION_OF_PEPTIDYL_SERINE_PHOSPHORYLATION
GOBP_NEUTROPHIL_HOMEOSTASIS
GOCC_CYTOSOLIC_RIBOSOME
GOMF_SERINE_TYPE_ENDOPEPTIDASE_INHIBITOR_ACTIVITY
Running Enrichment Score
Ranked List Metric
Rank in Ordered Dataset

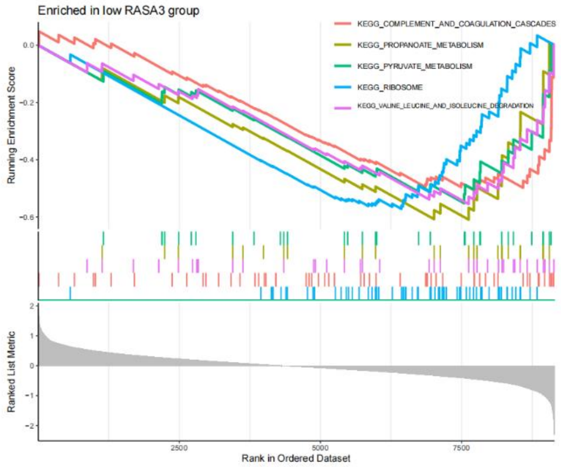

Enriched in low RASA3 group
KEGG_COMPLEMENT_AND_COAGULATION_CASCADES
KEGG_PROPANOATE_METABOLISM
KEGG_PYRUVATE_METABOLISM
KEGG_RIBOSOME
KEGG_VALINE_LEUCINE_AND_ISOLEUCINE_DEGRADATION
Running Enrichment Score
Ranked List Metric
Rank in Ordered Dataset

RNGTT

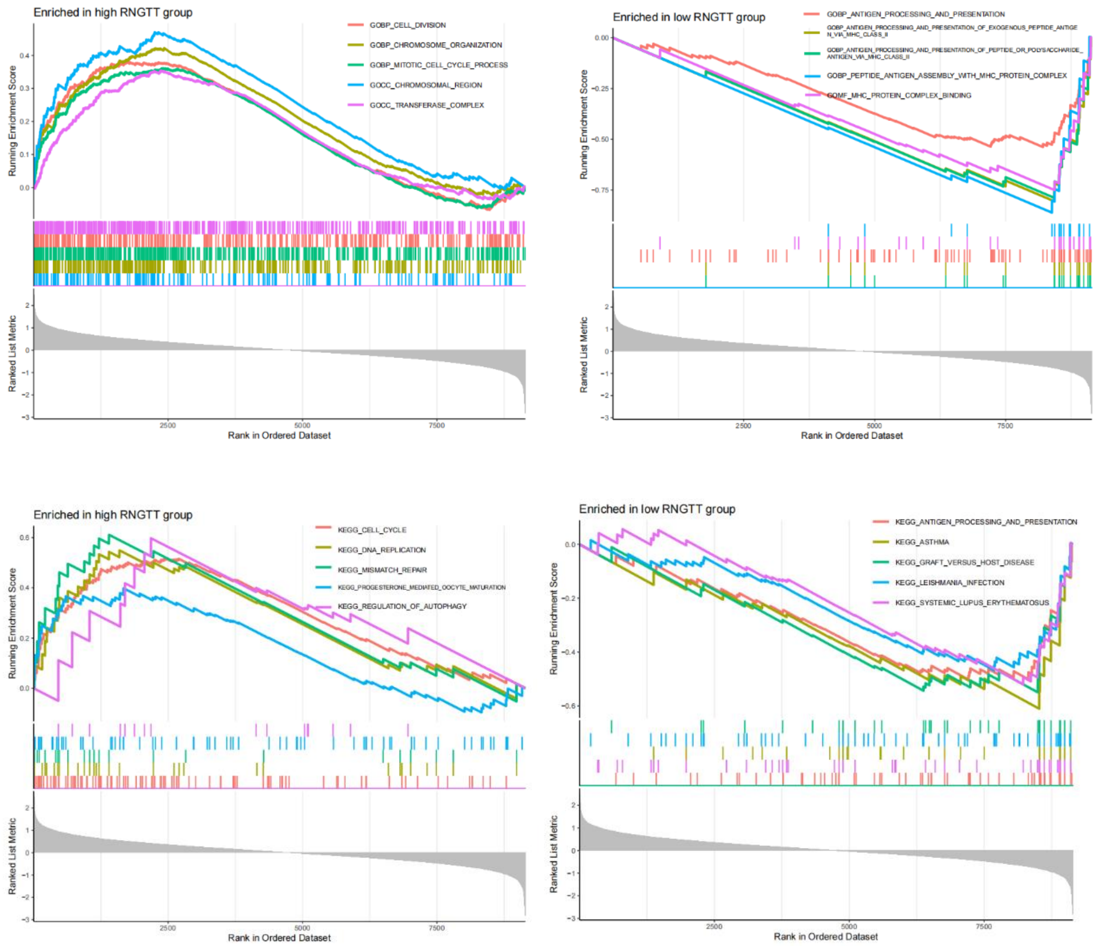

Enriched in high RNGTT group
GOBP_CELL_DIVISION
GOBP_CHROMOSOME_ORGANIZATION
GOBP_MITOTIC_CELL_CYCLE_PROCESS
GOCC_CHROMOSOMAL_REGION
GOCC_TRANSFERASE_COMPLEX
Running Enrichment Score
Ranked List Metric
Rank in Ordered Dataset
Enriched in low RNGTT group
GOBP_ANTIGEN_PROCESSING_AND_PRESENTATION
GOBP_ANTIGEN_PROCESSING_AND_PRESENTATION_OF_EXOGENOUS_PEPTIDE_ANTIGEN_VIA_MHC_CLASS_II
GOBP_ANTIGEN_PROCESSING_AND_PRESENTATION_OF_PEPTIDE_OR_POLYSACCHARIDE_ANTIGEN_VIA_MHC_CLASS_II
GOBP_PEPTIDE_ANTIGEN_ASSEMBLY_WITH_MHC_PROTEIN_COMPLEX
GOMF_MHC_PROTEIN_COMPLEX_BINDING
Running Enrichment Score
Ranked List Metric
Rank in Ordered Dataset
Enriched in high RNGTT group
KEGG_CELL_CYCLE
KEGG_DNA_REPLICATION
KEGG_MISMATCH_REPAIR
KEGG_PROGESTERONE_MEDIATED_OOCYTE_MATURATION
KEGG_REGULATION_OF_AUTOPHAGY
Running Enrichment Score
Ranked List Metric
Rank in Ordered Dataset
Enriched in low RNGTT group
KEGG_ANTIGEN_PROCESSING_AND_PRESENTATION
KEGG_ASTHMA
KEGG_GRAFT_VERSUS_HOST_DISEASE
KEGG_LEISHMANIA_INFECTION
KEGG_SYSTEMIC_LUPUS_ERYTHEMATOSUS
Running Enrichment Score
Ranked List Metric
Rank in Ordered Dataset

TSPAN4

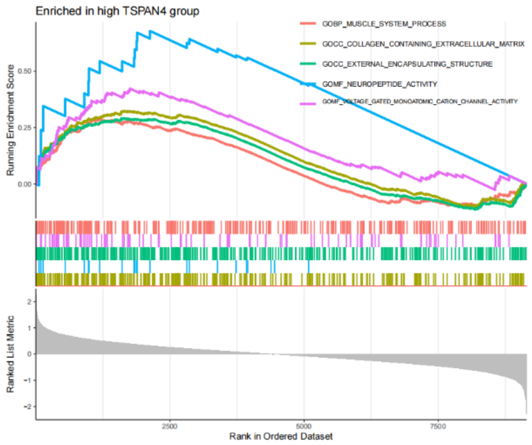

Enriched in high TSPAN4 group
GOBP_MUSCLE_SYSTEM_PROCESS
GOCC_COLLAGEN_CONTAINING_EXTRACELLULAR_MATRIX
GOCC_EXTERNAL_ENCAPSULATING_STRUCTURE
GOMF_NEUROPEPTIDE_ACTIVITY
GOMF_VOLTAGE_GATED_MONOATOMIC_CATION_CHANNEL_ACTIVITY
Running Enrichment Score
0.50
0.25
0.00
Ranked List Metric
2
1
0
-1
-2
2500
5000
7500
Rank in Ordered Dataset

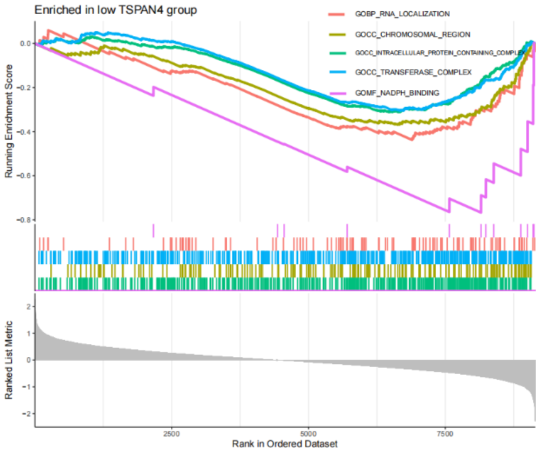

Enriched in low TSPAN4 group
GOBP_RNA_LOCALIZATION
GOCC_CHROMOSOMAL_REGION
GOCC_INTRACELLULAR_PROTEIN_CONTAINING_COMPLEX
GOCC_TRANSFERASE_COMPLEX
GOMF_NADPH_BINDING
Running Enrichment Score
0.0
-0.2
-0.4
-0.6
-0.8
Ranked List Metric
2
1
0
-1
-2
2500
5000
7500
Rank in Ordered Dataset

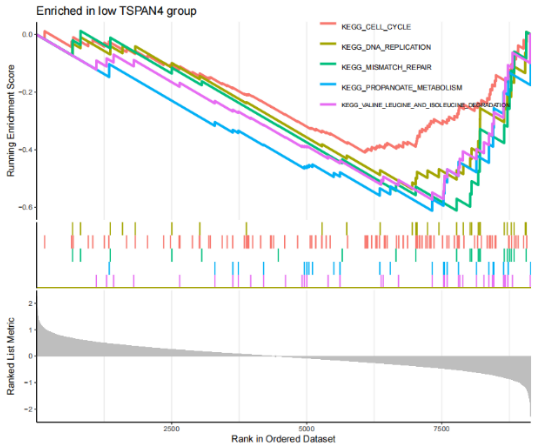

Enriched in low TSPAN4 group
KEGG_CELL_CYCLE
KEGG_DNA_REPLICATION
KEGG_MISMATCH_REPAIR
KEGG_PROPANOATE_METABOLISM
KEGG_VALINE_LEUCINE_AND_ISOLEUCINE_DEGRADATION
Running Enrichment Score
0.0
-0.2
-0.4
-0.6
Ranked List Metric
2
1
0
-1
-2
2500
5000
7500
Rank in Ordered Dataset

YTHDC2

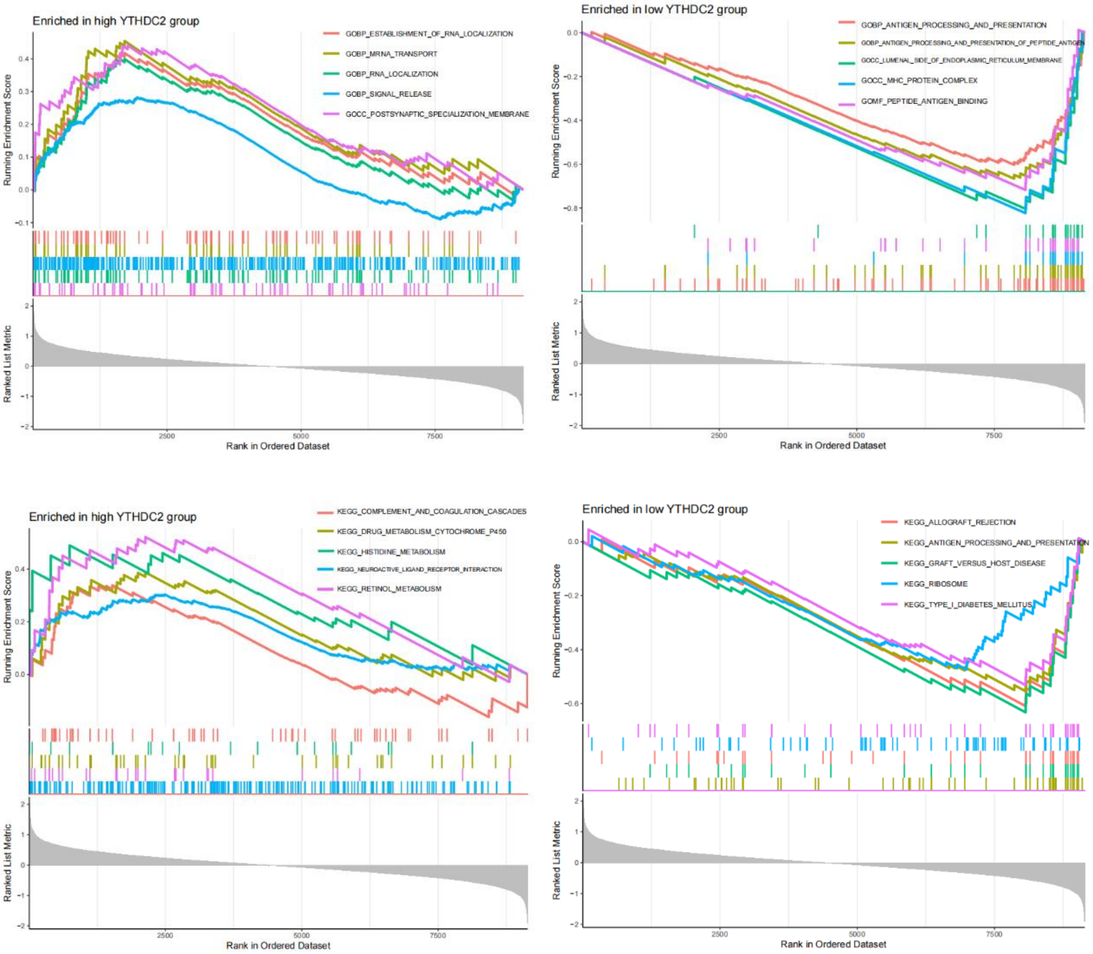

Enriched in high YTHDC2 group
GOBP_ESTABLISHMENT_OF_RNA_LOCALIZATION
GOBP_MRNA_TRANSPORT
GOBP_RNA_LOCALIZATION
GOBP_SIGNAL_RELEASE
GOCC_POSTSYNAPTIC_SPECIALIZATION_MEMBRANE
Enriched in low YTHDC2 group
GOBP_ANTIGEN_PROCESSING_AND_PRESENTATION
GOCC_MHC_PROTEIN_COMPLEX
GOMF_PEPTIDE_ANTIGEN_BINDING
Enriched in high YTHDC2 group
KEGG_COMPLEMENT_AND_COAGULATION_CASCADES
KEGG_DRUG_METABOLISM_CYTOCHROME_P450
KEGG_HISTIDINE_METABOLISM
KEGG_NEUROACTIVE_LIGAND_RECEPTOR_INTERACTION
KEGG_RETINOL_METABOLISM
Enriched in low YTHDC2 group
KEGG_ALLOGRAFT_REJECTION
KEGG_ANTIGEN_PROCESSING_AND_PRESENTATION
KEGG_GRAFT_VERSUS_HOST_DISEASE
KEGG_RIBOSOME
KEGG_TYPE_I_DIABETES_MELLITUS
Running Enrichment Score
Ranked List Metric
Rank in Ordered Dataset

ZFP37

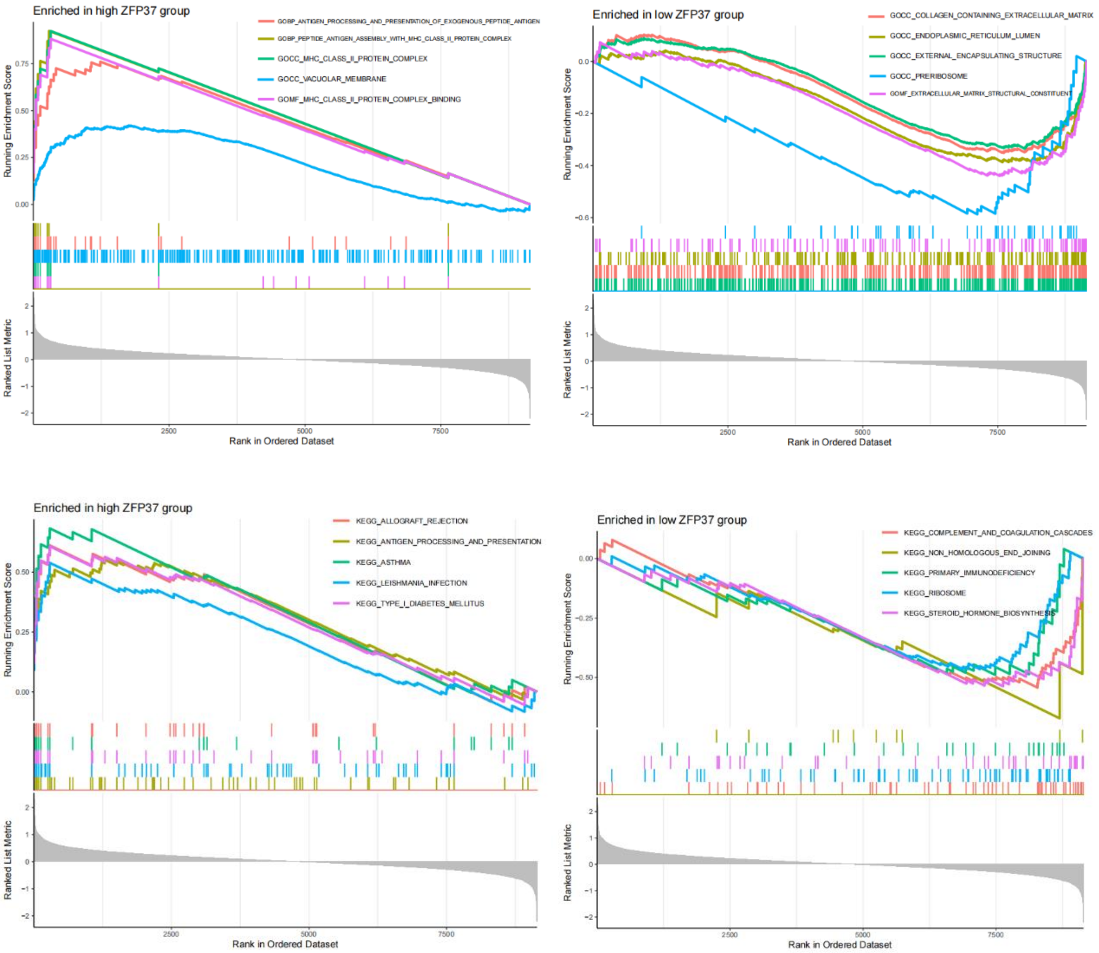
Enriched in high ZFP37 group
GOBP_ANTIGEN_PROCESSING_AND_PRESENTATION_OF_EXOGENOUS_PEPTIDE_ANTIGEN
GOBP_PEPTIDE_ANTIGEN_ASSEMBLY_WITH_MHC_CLASS_II_PROTEIN_COMPLEX
GOCC_MHC_CLASS_II_PROTEIN_COMPLEX
GOCC_VACUOLAR_MEMBRANE
GOMF_MHC_CLASS_II_PROTEIN_COMPLEX_BINDING
Running Enrichment Score
Ranked List Metric
Rank in Ordered Dataset
Enriched in low ZFP37 group
GOCC_COLLAGEN_CONTAINING_EXTRACELLULAR_MATRIX
GOCC_ENDOPLASMIC_RETICULUM_LUMEN
GOCC_EXTERNAL_ENCAPSULATING_STRUCTURE
GOCC_PRERIBOSOME
GOMF_EXTRACELLULAR_MATRIX_STRUCTURAL_CONSTITUENT
Running Enrichment Score
Ranked List Metric
Rank in Ordered Dataset
Enriched in high ZFP37 group
KEGG_ALLOGRAFT_REJECTION
KEGG_ANTIGEN_PROCESSING_AND_PRESENTATION
KEGG_ASTHMA
KEGG_LEISHMANIA_INFECTION
KEGG_TYPE_I_DIABETES_MELLITUS
Running Enrichment Score
Ranked List Metric
Rank in Ordered Dataset
Enriched in low ZFP37 group
KEGG_COMPLEMENT_AND_COAGULATION_CASCADES
KEGG_NON_HOMOLOGOUS_END_JOINING
KEGG_PRIMARY_IMMUNODEFICIENCY
KEGG_RIBOSOME
KEGG_STEROID_HORMONE_BIOSYNTHESIS
Running Enrichment Score
Ranked List Metric
Rank in Ordered Dataset
