# Supplementary material for: Integration of eQTL and GEO Datasets to Identify Genes Associated with Breast Ductal Carcinoma In Situ
Source: Curr Issues Mol Biol. 2025 Sep 11;47(9):747. doi: 10.3390/cimb47090747 (PMC12468441; doi:10.3390/cimb47090747)

Supplemental Figures S3. Principal component analysis (PCA) plots of gene expression profiles across different datasets before and after batch correction. Each symbol represents samples from distinct GEO datasets: GSE16873, GSE21422, GSE59246 and GSE7882.

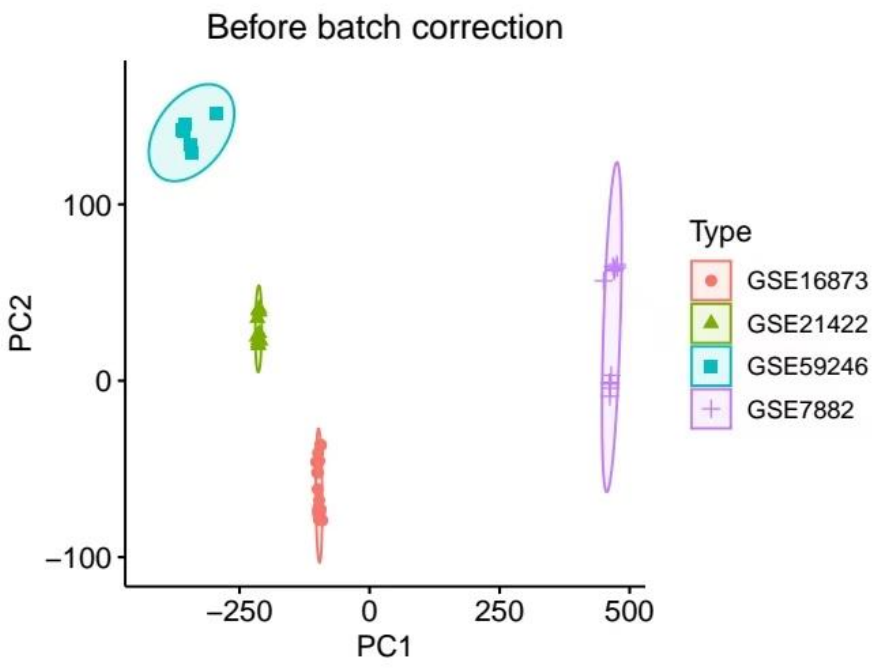

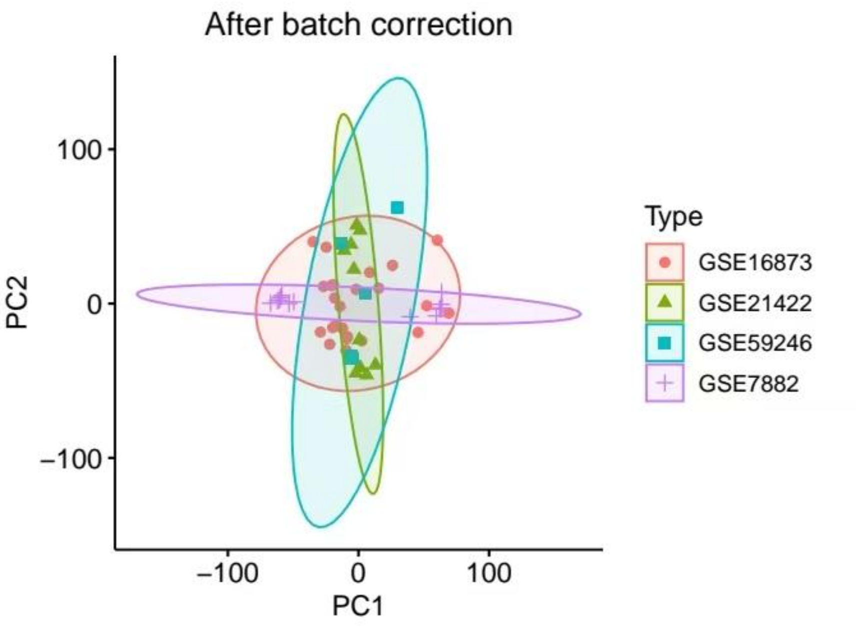

Supplement: Supplementary file 1 [file cimb-47-00747-s001.zip › Supplementary Figures S3.pdf]
